# MAGI1 Prevents Senescence and Promotes the DNA Damage Response in ER^+^ Breast Cancer

**DOI:** 10.3390/cells12151929

**Published:** 2023-07-25

**Authors:** Janine Wörthmüller, Simona Disler, Sylvain Pradervand, François Richard, Lisa Haerri, Gustavo A. Ruiz Buendía, Nadine Fournier, Christine Desmedt, Curzio Rüegg

**Affiliations:** 1 Laboratory of Experimental and Translational Oncology, Department of Oncology, Microbiology and Immunology (OMI), Faculty of Science and Medicine, University of Fribourg, 1700 Fribourg, Switzerland; 2 Lausanne Genomic Technologies Facility (LGTF), University of Lausanne, 1015 Lausanne, Switzerland; 3 Laboratory for Translational Breast Cancer Research, KU Leuven, 3000 Leuven, Belgium; 4 Translational Data Science-Facility, AGORA Cancer Research Center, Swiss Institute of Bioinformatics (SIB), Bugnon 25A, 1005 Lausanne, Switzerland

**Keywords:** breast cancer, MAGI1, senescence, DNA damage, DNA repair, PI3K/AKT signaling, MAPK signaling, HDAC, PARP1

## Abstract

MAGI1 acts as a tumor suppressor in estrogen receptor-positive (ER^+^) breast cancer (BC), and its loss correlates with a more aggressive phenotype. To identify the pathways and events affected by MAGI1 loss, we deleted the MAGI1 gene in the ER^+^ MCF7 BC cell line and performed RNA sequencing and functional experiments in vitro. Transcriptome analyses revealed gene sets and biological processes related to estrogen signaling, the cell cycle, and DNA damage responses affected by MAGI1 loss. Upon exposure to TNF-α/IFN-γ, MCF7 MAGI1 KO cells entered a deeper level of quiescence/senescence compared with MCF7 control cells and activated the AKT and MAPK signaling pathways. MCF7 MAGI1 KO cells exposed to ionizing radiations or cisplatin had reduced expression of DNA repair proteins and showed increased sensitivity towards PARP1 inhibition using olaparib. Treatment with PI3K and AKT inhibitors (alpelisib and MK-2206) restored the expression of DNA repair proteins and sensitized cells to fulvestrant. An analysis of human BC patients’ transcriptomic data revealed that patients with low MAGI1 levels had a higher tumor mutational burden and homologous recombination deficiency. Moreover, MAGI1 expression levels negatively correlated with PI3K/AKT and MAPK signaling, which confirmed our in vitro observations. Pharmacological and genomic evidence indicate HDACs as regulators of MAGI1 expression. Our findings provide a new view on MAGI1 function in cancer and identify potential treatment options to improve the management of ER^+^ BC patients with low MAGI1 levels.

## 1. Introduction

Breast cancer (BC) is the most commonly diagnosed cancer; it is the second leading cause of cancer-related deaths in women worldwide [1], where it is estimated that about one out of eight women will develop invasive BC over their lifetime [2]. More than 70% of all BCs are classified as estrogen receptor-positive (ER^+^) and are treated using hormonal therapy alone or in combination with other modalities [3]. One important problem is that 30–50% of treated women develop resistance towards estrogen inhibition therapy [4]; hyperactivation of the phosphoinositide 3-kinase (PI3K) signaling pathway has been described as one of the causes of this resistance [5].

Cellular senescence is defined as a stable and long-term loss of proliferative capacity [6], while quiescence is a reversible growth/proliferation arrest [7]; given sufficient growth stimulation, quiescent cells can eventually reenter the cell cycle. Most antitumor therapies target highly proliferative cancer cells, while senescent or slow-proliferative dormant/quiescent cancer cells are not affected, can persist, and can eventually cause tumor relapse (reviewed in [8]). Quiescence and senescence can be triggered in response to various intrinsic and extrinsic stimuli, such as persistent DNA damage and mutations, oncogene activation, tumor suppressor loss, oxidative and genotoxic stresses, radiation, or chemotherapeutic agents. The two main pathways that regulate these processes are the p53/p21^WAF1/CIP1^ and p16^INK4A^/RB pathways, which are also robustly modulated by the p38/MAPK and PI3K/AKT/mTOR signaling pathways (reviewed in [9]).

Many DNA lesions occur daily as a result from either endogenous cellular processes or exogenous challenges, including ionizing radiation (IR), ultraviolet (UV) light, or chemotherapeutic agents [10]. Double-strand breaks (DSBs) are the most lethal type of DNA lesion which, if left unrepaired, can cause genomic instability that favors tumor initiation or progression [11]. To counteract these lesions, cells have developed an efficient system to sense and repair damaged DNA. The DNA damage repair (DDR) pathway consists of a complex network of cell cycle progression checkpoints as well as multiple DNA repair pathways. A defective DDR not only promotes the initiation of cancer, but it also allows the tumor cells to quickly acquire additional mutations that favor cancer progression [12].

Overactivation of the oncogenic PI3K/AKT signaling pathway contributes to increased cell survival and chemoresistance, and it has been observed in most solid tumors, including in BC [13]. Emerging evidence suggests that the PI3K/AKT pathway can also regulate DDR, possibly through the suppression of the two main DDR pathways [14], i.e., homologous recombination (HR) and nonhomologous DNA end joining (NHEJ), providing a time window that allows cells to accumulate mutations while escaping cell death. Thus, the PI3K/AKT pathway represents an attractive target, especially in BCs that are resistant to conventional therapies. Similarly, inhibitors of poly (ADP-ribose) polymerase-1 (PARP1), an enzyme that plays an important role in the DNA damage response, have been developed as therapeutic anti-cancer agents. Their use has resulted in synthetic lethality in HR-deficient tumor cells [15], and they have been approved as a maintenance treatment in patients with advanced ovarian cancer or as an adjuvant treatment for patients with germline *BRCA-1/2*-mutated BCs [16].

MAGI1 (membrane-associated guanylate kinase, WW, and PDZ domain-containing protein 1), a cytoplasmic scaffolding protein with tumor suppressor functions, is lost or has its expression decreased in several cancers, including BC (reviewed in [17]). Recent publications have shown that MAGI1 is able to modulate the activity of oncogenic pathways such as the PI3K/AKT, Wnt/β-catenin, and MAPK/ERK signaling pathways [18,19,20,21,22,23]. In addition, MAGI1 loss is associated with the acquired resistance to hormonal therapy in ER^+^ BC: low MAGI1 levels predict a more aggressive behavior, whereas high levels correlate with a lower risk of relapse in ER^+^HER2^−^ patients treated with tamoxifen compared with those who are untreated or who are chemotherapy-treated [21]. Different mechanisms such as mechanical stress, estrogen signaling, or inflammation have been described to regulate MAGI1 expression (reviewed in [17]). However, the exact mechanisms that contribute to MAGI1 downregulation in BC cells, including epigenetic mechanisms, largely remain to be elucidated.

To characterize the tumor suppressive pathways and cellular events regulated by MAGI1, we deleted the MAGI1 gene in MCF7 cells and performed transcriptomics, cellular, and functional studies. Our results demonstrate a role of MAGI1 in protecting cells against senescence and DNA damage, which involves the PI3K/AKT and MAPK signaling pathways. We also show that MAGI1 loss sensitizes cells to PARP1 inhibitors and PI3K/AKT-targeted therapies. Additionally, we prove that *MAGI1* mRNA levels are upregulated after treatment with histone deacetylase (HDAC) inhibitors in different BC cell lines, which suggests that histone deacetylation could be part of the mechanisms that downregulate MAGI1 during tumor progression. In view of the increased aggressiveness of the ER^+^ BC subtype with low MAGI1 levels, these new results provide unanticipated opportunities to explore novel potential therapeutic strategies for this subgroup of BC patients.

## 2. Materials and Methods

### 2.1. Cell Culture

The human cell line HEK293T and human BC cell lines MCF7, MDA-MB-231, and BT-474 were obtained from the American Type Culture Collection (ATCC; Rockville, MD, USA). Cells were cultured in Dulbecco’s modified Eagle’s medium (DMEM; Cat. No. p04-04500, PAN-Biotech, Bayern, Germany), which was supplemented with 10% fetal bovine serum (FBS; Cat. No. P40-37500, PAN-Biotech) and 1% penicillin/streptomycin solution (Gibco|Thermo Fisher Scientific, Waltham, MA, USA) and maintained at 37 °C in a humidified 5% CO_2_ atmosphere.

### 2.2. Design of MAGI1 KO CRISPR Constructs and LV Production

Individual single guided RNAs (sgRNA) targeting the PDZO region of MAGI1 were designed using the Integrated DNA Technologies (IDT) website. The following sequences were used: MAGI1_gRNA_PDZ0_frw1, 5′-CACCGCACGTCATAGCGGGGCAAGC-3′; MAGI1_gRNA_PDZ0_rev1, 5′-AAACGCTTGCCCCGCTATGACGTGC-3′ and MAGI1_gRNA_PDZ0_frw2, 5′-CACCGTCTGACGGCCTTGAAGGTGA-3′; and MAGI1_gRNA_PDZ0_rev2, 5′-AAACTCACCTTCAAGGCCGTCAGAC-3′. The sequences were cloned into the lentiCRISPR v2 sequencing plasmid (Addgene plasmid #52961) as described in [24]. All of the constructed plasmids were confirmed by restriction enzyme digestion and DNA sequencing (Microsynth, Balgach, Switzerland). Lentivirus particles were produced as follows: HEK293T cells were co-transfected by the CaPO_4_ method with 3 µg of the envelope plasmid pMD2.G-VSVG (Addgene plasmid #12259), 8 µg of the packaging plasmid psPAX2 (Addgene plasmid #12260), and 10 µg of the transfer plasmid (MAGI1_KO_A; MAGI1_KO_B; EMPTY_VECTOR). Lentiviruses in the supernatant of HEK293T cells were harvested 48 and 72 h after transfection and were filtered (0.45 μm).

### 2.3. RNA Sequencing, Data Processing, and Enrichment Analysis

Five independent samples from each construct were prepared and sent for RNA sequencing at the Lausanne Genomics Technologies Facility (GTF, UNIL) in Lausanne, Switzerland. Samples were normalized for 1 μg of RNA in a volume of 20 μL and were sequenced using the NextSeq500 sequencer (Illumina, San Diego, CA, USA). For sequencing data processing and quality control, purity-filtered reads were quality-trimmed using Cutadapt [25] (v. 2.5). Reads that matched ribosomal RNA sequences were removed using fastq_screen [26] (v. 0.11.1). The remaining reads were further filtered for low complexity using the reaper tool [27] (v. 15-065). Reads were aligned against the Homo sapiens.GRCh38.102 genome using STAR [28] (v. 2.5.3a). The number of read counts per gene locus was summarized using htseq-count [29] (v. 0.9.1), and we used Homo sapiens.GRCh38.102 as the gene annotation. The quality of the RNA-seq data alignment was assessed using RSeQC [30] (v. 2.3.7). A statistical analysis was performed using read counts per gene locus (R v. 4.0.3). Genes with low counts were filtered out according to the rule of 1 count per million (cpm) in at least one sample. Library sizes were scaled using TMM normalization [31] (v. 3.18.1) and were log-transformed using the limma cpm function with parameter prior.count = 1 [32] (v. 3.32.2). The sequencing batch effect was removed using the Combat tool [33]. A differential expression was computed using the limma [34] on 14245 filtered genes. The contrasts of KO_A vs. VEC and KO_B vs. VEC were extracted from the model. *p*-values were adjusted for multiple testing using the Benjamini–Hochberg method for each comparison separately. A GO terms enrichment analysis was performed using the enrichGO function from clusterProfiler [35] (v. 3.12.0). Up- and down-regulated protein-coding genes were selected as genes with an adjusted *p*-value <0.05 in both conditions (KO_A and KO_B). The significant GO terms were processed for redundant terms using the ‘simplify’ function from clusterProfiler. Enrichment of MSigDB hallmark gene sets (https://www.gsea-msigdb.org/gsea/msigdb/ (accessed on the 18 June 2021)) was tested with the ‘enricher’ function from clusterProfiler.

### 2.4. Chemicals and Reagents

Recombinant Human TNF-α (Cat. No. 300-01A), IFN-γ (Cat. No. 300-02), and insulin growth factor (IGF; Cat. No. 100-11) were purchased from PeproTech (Cranbury, NJ, USA). Sodium butyrate (Cat. No. S1200) and cisplatin (Cat. No. C2210000) were purchased from Sigma-Aldrich (Saint Louis, MO, USA). The inhibitors CUCD-101 (Cat. No. HY-10223), decitabine (HY-A0004), santacruzmate A (HY-N0931), zebularine (HY-13420), olaparib (HY-10162), alpelisib (HY-15244), and MK-2206 (HY-108232) were all purchased from MedChemExpress (Sollentuna, Sweden). Fulvestrant (ICI 182,780; Cat. No. 1047) was obtained from Tocris Bioscience (Bristol, UK). A medium containing 0.5% DMSO was used as a vehicle-only control for the experiments.

### 2.5. Antibodies

The following antibodies were used in this study: Anti-MAGI1 (1:1′000; Cat. No. M5691, Sigma-Aldrich), anti-GAPDH (1:5′000; Cat. No. G9545, Sigma-Aldrich), anti-gamma H2A.X (phospho Ser139) (1:1′000, Cat. No. ab26350, Abcam, Cambridge, UK), anti-AKT (Cat. No. 9272), anti-phospho AKT (S473) (Cat. No. 4060), anti-p44/42 MAPK (Erk1/2) (Cat. No. 9102), anti-phospho p44/42 MAPK (Erk1/2) (Thr202/Tyr204) (Cat. No. 9101), anti-p38 MAPK (Cat. No. 9212), anti-phospho p38 MAPK (Thr180/Tyr182) (Cat. No. 9211), anti-p21 (Cat. No. 2946), anti-PTEN (Cat. No. 9552), anti-mTOR (Cat. No. 2972), and anti-phospho mTOR (Ser2448) (Cat. No. 2971), all acquired from Cell Signaling Technology (Danvers, MA, USA) and used at a dilution of 1:1′000. Secondary goat anti-rabbit- or anti-mouse (HRP)-labeled antibodies (Cat. No. A0545 and A5278) were obtained from Sigma-Aldrich and used at a dilution of 1:10′000. To assess the different proteins involved in repair pathways, the double strand breaks (DSBs) Repair Antibody Sampler Kit was used (Cat. No. 9653, Cell Signaling Technology).

### 2.6. Cell Treatments

MCF7 cells were treated with 20 ng/μL of TNF-α and 33 ng/μL of IFN-γ (high concentration) or 10 ng/μL of TNF-α and 15 ng/μL of IFN-γ (medium concentration) for a maximum of 48 h, and then subjected to protein extraction for a Western blot analysis or to senescence-associated β-Galactosidase staining. Low concentrations of TNF-α and IFN-γ (0.5 ng/μL and 0.75 ng/μL, respectively) were used for RNA extraction followed by quantitative real-time PCR (qPCR) or were used for long-term monitoring using the Incucyte™ live cell imaging system (Essen Bioscience Inc., Ann Arbor, MI, USA). Images and growth curves were evaluated using the IncuCyte™ software system (v. 2018A, Essen Bioscience). IGF was added at a concentration of 25 nM after 250 h of treatment with a low concentration of TNF-α and IFN-γ. For measuring cell viability after irradiation, 2′000–5′000 cells/well were seeded into 96-well plates (Corning; Sigma-Aldrich), and a single 30 Gy-dose was applied by using an X-ray unit (X-RAD iR225 Biological Irradiator, North Branford, CT, USA) operated at a 125 kV and 20 mA while using a 2 mm Al filter. Cells were further maintained for 48–144 h for cell viability assays. For Western blot analyses, cells were seeded in 75 cm^2^ flasks (Corning; Sigma-Aldrich), which were irradiated with a 30 Gy-dose as above. In experiments with irradiation in combination with alpelisib or MK-2206, cells were treated 1 h after irradiation with a concentration of 2 μM of alpelisib and 6 μM of MK-2206, respectively. For olaparib and cisplatin treatments, cells were treated for 48 h with 60 μM of cisplatin alone or with 15 μM of cisplatin in combination with 10 μM of olaparib; the cells were subjected to cell monitoring (using the Incucyte™ system) or to a Western blot analysis. For treatments using alpelisib and fulvestrant, cells were treated for 72 h with 2 μM of alpelisib alone or in combination with 0.5 μM of fulvestrant followed by cell monitoring or cell viability assays. For experiments using HDAC and DNMT inhibitors, 3 × 10^5^ cells/well were seeded in 6-well plates (Corning; Sigma-Aldrich) and then treated for 24 h or 48 h with the inhibitors at the following concentrations: 2.5 mM of NaBt, 0.5 μM of CUCD-101, and 10 μM of decitabine in the case of MCF7 and BT-474 cells; and 5 mM of NaBt, 0.1 μM of CUCD-101, and 100 μM of decitabine in the case of MDA-231 cells. For santacruzmate A, MCF7 cells were treated with a concentration of 10 μM for 24 h. For zebularine, MCF7 cells were treated with a concentration of 150 μM, whereas MDA-231 and BT-474 cells were treated with a concentration of 100 μM. After the treatments, total RNA was extracted followed by a qPCR analysis. Differences between treatments in MCF7 VEC and MCF7 MAGI1 KO cells were analyzed for significance using a two-way ANOVA test with Sidak’s multiple comparison being used as a post hoc test.

### 2.7. Senescence-Associated β-Galactosidase Staining of Cultured Cells

Cells were treated with a combined concentration of 10 ng/μL of TNF-α and 15 ng/μL of IFN-γ for 48 h. Thereafter, cells were washed with PBS and treated with the senescence β-gal staining kit (Cat. No. 9860, Cell Signaling Technology) according to the manufacturer’s instructions. Briefly, cells were fixed with the fixative solution for 15 min at room temperature, washed with PBS, stained with the β-Galactosidase staining solution, and incubated for 16–20 h at 37 °C. The next day, cells were washed with PBS and observed under a bright field microscope for blue-colored stained cells. Pictures were taken and blue-stained cells were counted.

### 2.8. Cell Viability (MTT Assay)

The MTT assay was used to assess the sensitivity of the cells to irradiation, alpelisib, fulvestrant, and cisplatin treatments. Cell viability after treatments were measured by adding MTT (Sigma-Aldrich) at a concentration of 0.5 mg/mL during 3 h and by using DMSO to dissolve the purple formazan crystals. The absorbance was measured at 570 nm using a spectrophotometer (TECAN infinite M200PRO, Männedorf, Switzerland). Experiments were performed in triplicate.

### 2.9. Real-Time Reverse Transcription PCR (RT-qPCR)

RNA was extracted using the Macherey-Nagel™ NucleoSpin™ RNA Plus Kit (Cat. No. 15370195, Thermo Fisher Scientific). An amount of 1 µg of RNA was retro-transcribed to cDNA using the High-Capacity cDNA Reverse Transcription Kit (Cat. No. 4368814, Thermo Fisher Scientific). A qPCR analysis was performed using the SensiFASTTM SYBR Hi-ROX kit (Cat. No. BIO-92020) in a StepOnePlus thermocycler (Applied Biosystems, Foster City, CA, USA). The following thermal profile was applied: 1 cycle at 95 °C for 2 min, 40 cycles at 95 °C for 5 s, and 60 °C for 18 s. Differences in fold expression were calculated according to the 2^−ΔΔCt^ method. The list of primers used can be found in the table below:


**Gene**

**Forward Primer (5′-3′)**

**Reverse Primer (5′-3′)**

*MAGI1*
TTCAAGGCCGTCAGACAAATGGGGGTAAAGGTTATCCC
*GAPDH*
GGACCTGACCTGCCGTCTAGCCACCACCCTGTTGCTGTAG
*P21*
GATTCGGGATATGCTGTTGGGTTCTGAGCTGGCACAGTGA
*P27*
GGTTAGCGGAGCAATGCGTCCACAGAACCGGCATTTG
*E2F1*
GGGGAGAAGTCACGCTATGACTCAGGGCACAGGAAAACAT
*YWHAZ*
ACTTTTGGTACATTGTGGCTTCAACCGCCAGGACAAACCAGTAT

### 2.10. Western Blotting

Cells were collected using a cell scraper and protein was extracted using a RIPA buffer (Cell Signaling Technology) containing a protease inhibitor cocktail (Cat. No. P8340, Sigma-Aldrich) and 1 mM of sodium orthovanadate (Na_3_VO_4_; Sigma). Protein concentrations were determined using the BC assay (BC Assay Protein Quantitation Kit, Uptima, Interchim, Montluçon, France) and equal amounts of protein (40 μg) were separated by NuPAGE™ 3 to 8% Tris-Acetate or Bolt™ 4 to 12% Bis-Tris mini protein gels (Cat. No. EA03752BOX and NW04122BOX, Thermo Fisher Scientific) and transferred onto nitrocellulose membranes using the Trans-Blot Turbo Transfer System (Bio-Rad, Hercules, CA, USA). Membranes were blocked with 5% BSA (Sigma-Aldrich) in TBS-Tween for 1 h at room temperature and incubated overnight at 4 °C with the primary antibodies being diluted in 2% BSA. The following day, the membranes were incubated with secondary goat anti-rabbit- or anti-mouse (HRP)-labeled antibodies. Signals were detected using Immobilon Luminata Forte (Cat. No. WBLUF0500, Millipore; Sigma-Aldrich) in the iBright CL1000 imaging system (Thermo Fisher Scientific). A densitometric analysis of phosphorylated proteins was performed using Alpha View software (v. 3.5.0.927) and was normalized to the expression of GAPDH. The treatments and corresponding Western blots were performed a minimum of 3 times.

### 2.11. Comet Assay

Cells were irradiated with a 30 Gy-dose as described above and analyzed for DNA fragmentation after 48 h using a comet assay kit (Cat. No. ab238544, Abcam). Briefly, the comet slides were coated with 35 μL of agarose to form a base layer; then, 75 μL of a mixture of cell suspension at a density of ∼1 × 10^6^ cells/mL and agarose at a ratio of 1:5 was applied on top of the base layer. The comet slides were immersed for 1 h at 4 °C in a cold fresh lysis solution followed by incubation in an alkaline solution for 30 min at 4 °C according to the manufacturer instructions. Slides were placed in an electrophoresis tank pre-filled with cold Tris-borate-EDTA buffer (TBE) electrophoresis solution. Electrophoresis was performed at 30 V over 10–15 min. The slides were then rinsed twice with distilled water, immersed in ethanol 70% for 5 min, and incubated for 15 min using the Vista Green DNA Dye. The comets were viewed using an epifluorescence microscope (Leica, Microsystems, Renan, Switzerland) with a FITC filter, and images of 40–90 comets were collected for each group using a digital imaging system. All of the comet images were analyzed using the ImageJ software with the plug-in OpenComet [36] (v. 1.3). The extent of DNA strand breaks was expressed as the tail moment (tail moment = tail length × % of DNA in the tail) and olive moment (olive moment = (tail mean-head mean) × % of DNA in the tail). Superimposed comets and comets without distinct head (‘clouds’, ‘ghost cells’, or ‘hedgehogs’) were excluded from the analysis. An unpaired *T*-test (two-tailed) was used to analyze the differences between MCF7 VEC and MCF7 MAGI1 KO tail and olive moments, and the data are presented as mean ± SEM. Experiments were performed twice.

### 2.12. Transcriptomic Analysis of Human Patient’s Data

The ICGC dataset, including the clinical data, gene expression, tumor mutational burden (TMB), and homologous recombination deficiency (HRD) scores, was retrieved from the supplementary data of Nik-Zainal et al. [37]. The TCGA dataset [38], including the clinical data and normalized gene expression, was retrieved through the cBioportal on the 08/04/19 [39]. Subtypes were assessed according to the ER immunohistochemistry and HER2 immunohistochemistry or FISH status, where only patients with ER^+^HER2^−^ BC were retained. The number of patients included was 206, 207, and 290 for the HRD, TMB, and gene expression signatures, respectively. Gene expression signatures were retrieved from the literature (ESR1_signature, AURKA proliferation [40]; AKT_MTOR_HG [41]; AKT_MTOR_MG [42]; PIK3CA-GS-mut [43]; PTEN_loss [44]; beta_catenin.up [45]; MAPK.up [46]; MEK.up [47]; Wound.up [48]) and computed as described in [49]. Correlations were assessed using Spearman coefficients. In the heatmap, only significant correlations are colored: red is anti-correlated and blue is correlated. The analysis was performed using R 4.2.1.

### 2.13. Bioinformatic Analysis of Predicted Histone Acetylation Sites and HDAC2 Binding Partners Motifs along the MAGI1 Promoter Region

The binding sites of HDAC2-associated binding partners were identified using the FIMO tool [50] from the MEME suite of motif-based sequence analysis tools (v. 5.5.3) [51]. HDAC2-associated binding partners with DNA binding motifs were obtained from the human transcription factor binding model database called HOCOMOCO [52]. The MAGI1 regulatory region (2 kb up/downstream of the MAGI1 transcriptional start site) was analyzed for the occurrences of binding motifs for HDAC2-associated binding partners.

## 3. Results

### 3.1. Transcriptome Analyses Revealed Biological Processes and Pathways Related to Estrogen and mTOR Signaling, Cell Cycle, DNA Damage Checkpoints, and DNA Damage Response Being Altered in MCF7 MAGI1 KO Cells

**Figure 1:**
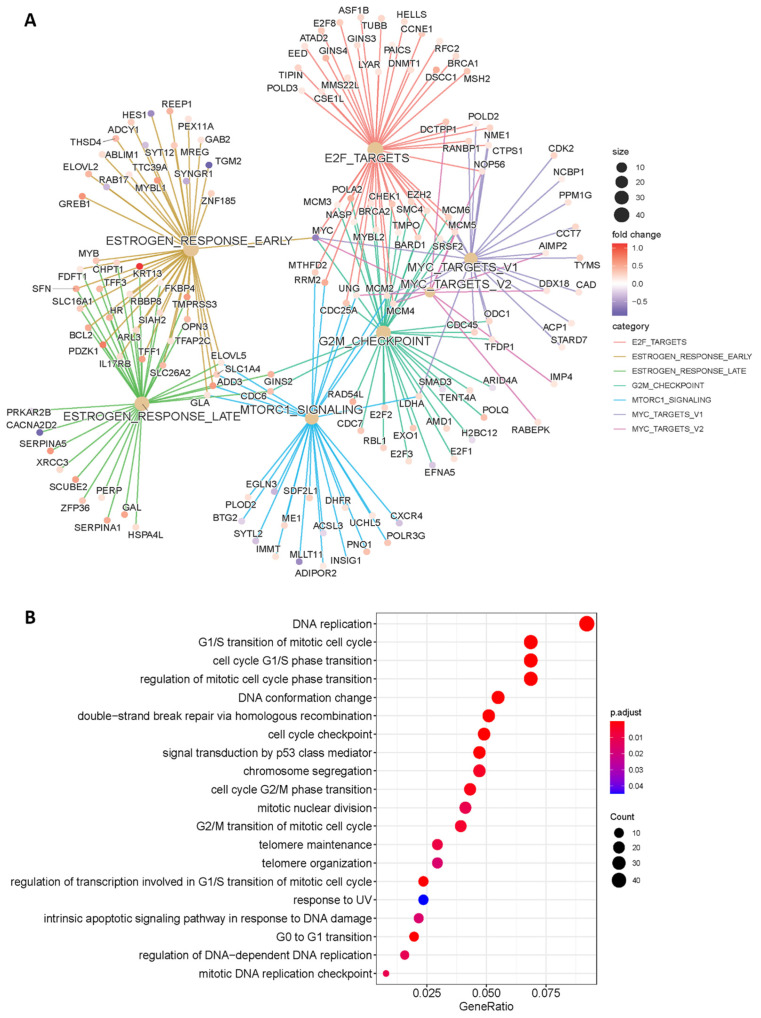
**Pathways and processes modulated by MAGI1 loss in MCF7 cells as revealed by transcriptomic analyses.** (**A**) Results of the enrichment analysis of gene sets from the MSigDB Hallmark database. Pathways and up- and down-regulated target genes that are significantly enriched by MAGI1 loss are represented. (**B**) Gene ontology (GO) analysis of the biological processes that are enriched in MCF7 MAGI1 KO cells compared with MCF7 VEC cells (the full list of the enriched biological processes can be found in Supplementary Table S3).

To identify genes and signaling pathways that are modulated by MAGI1 loss in ER^+^ BC cells, we decided to inactivate the MAGI1 gene in ER^+^HER2^−^ MCF7 cells using the CRISPR/Cas9 strategy. To this end, two independent MAGI1 CRISPR/Cas9 knockout (KO) constructs (named MAGI1 KO_A and MAGI1 KO_B) were designed to target the PDZO region of MAGI1 at different sites (Figure S1A), causing a frameshift deletion in the open reading frame (ORF) of the MAGI1 gene (for more information, see the Materials and Methods section). As clonal heterogeneity is important in tumor progression [53], we decided to perform a bulk selection of the transduced cells rather than a clonal selection [54]; the latter strategy would select individual clones, thereby eliminating the full heterogeneity of the parental population that they derive from [55]. MAGI1 mRNA and protein levels were reduced by approximately 50–60% in the selected bulk cell population (Figure S1B,C), which was similar in both constructs, and this reduction was consistently sustained along cell passages. High-throughput RNA-sequencing (RNA-Seq) of MCF7 MAGI1-low (MAGI1 CRISPR KO_A and MAGI1 CRISPR KO_B) vs. MCF7 MAGI1-high control cells (MAGI1 CRISPR VEC) revealed many differentially expressed genes (DEG) in MAGI1-high vs. MAGI1-low MCF7 cells (Supplementary Table S1). To perform the pathway enrichment analyses, we selected 548 up- and 463 down-regulated protein-coding genes that were modulated by both the MAGI1 CRISPR KO_A and MAGI1 CRISPR KO_B constructs (Supplementary Table S2). The enrichment analysis revealed gene sets that were related to estrogen and mTOR signaling and the cell cycle, predominantly the G2/M DNA damage checkpoint, MYC, and E2F targets (Figure 1A).

We identified *ESR1* and many estrogen-responsive genes and/or cell cycle regulators such as *MYC, TGM2, HES1, WNK4, CCDN1*, and *CCNE1* that were modulated in MCF7 MAGI1 KO cells. Other estrogen-responsive genes such as *MYBL1, PDZK1, KRT13,* and *GREB1* and genes encoding subunits of different DNA polymerases (*POLD3, POLD2, POLA2*); E2F transcription factor-related genes (*E2F1, E2F3, E2F2, E2F8, CHEK1*) that are involved in cell cycle progression, senescence, and DNA damage response [56]; and important cell cycle regulator genes (*CDC45*, *CDC7*, *CDC25A*) were also modulated in MCF7 MAGI1 KO cells. *BRCA1* and *BRCA2*, which are critical tumor suppressor genes involved in DNA repair [57], were also affected in MCF7 MAGI1 KO cells. Additionally, oncogenic signatures included the ERBB2, MTOR (PI3K/AKT), KRAS, RAF, and MEK (MAPK) signaling networks (Figure S2). The gene ontology (GO) enrichment analysis of the biological processes revealed affected gene sets that were related to the different phases of the cell cycle (G0, G1/S, G2/M) and the DNA damage checkpoint. Specifically, processes related to DNA replication, G1/S transition, regulation of the mitotic cell cycle phase transition, double-strand break repair via HR, response to UV, or G0 to G1 transition were significantly enriched in MCF7 MAGI1 KO cells (Figure 1B). The G0 to G1 transition is a relevant checkpoint to maintain cancer cells in dormancy [58], while the G2/M DNA damage checkpoint ensures that cells do not initiate mitosis until damaged DNA is repaired or DNA is completely replicated. Cells with a defective G2/M checkpoint will enter the M phase before repairing their DNA [59], which results in the accumulation of mutations.

These results indicate that reduced MAGI1 levels impact pathways that are related to estrogen and mTOR signaling, cell cycle, DNA damage checkpoints, and DNA damage response that were previously not associated with MAGI1. Due to these findings and the fact that MAGI1 downregulation was described to affect cell cycle progression by increasing the proliferation rate of MCF7 cells [21], we decided to study the role of MAGI1 during senescence in greater detail using the CRISPR_B construct, hereinafter termed MCF7 MAGI1 KO.

### 3.2. Combined TNF-α/IFN-γ Treatment Promotes a Deep Quiescence/Senescence Phenotype in MCF7 MAGI1 KO Cells and Activates the PI3K/AKT and MAPK Signaling Pathways

**Figure 2:**
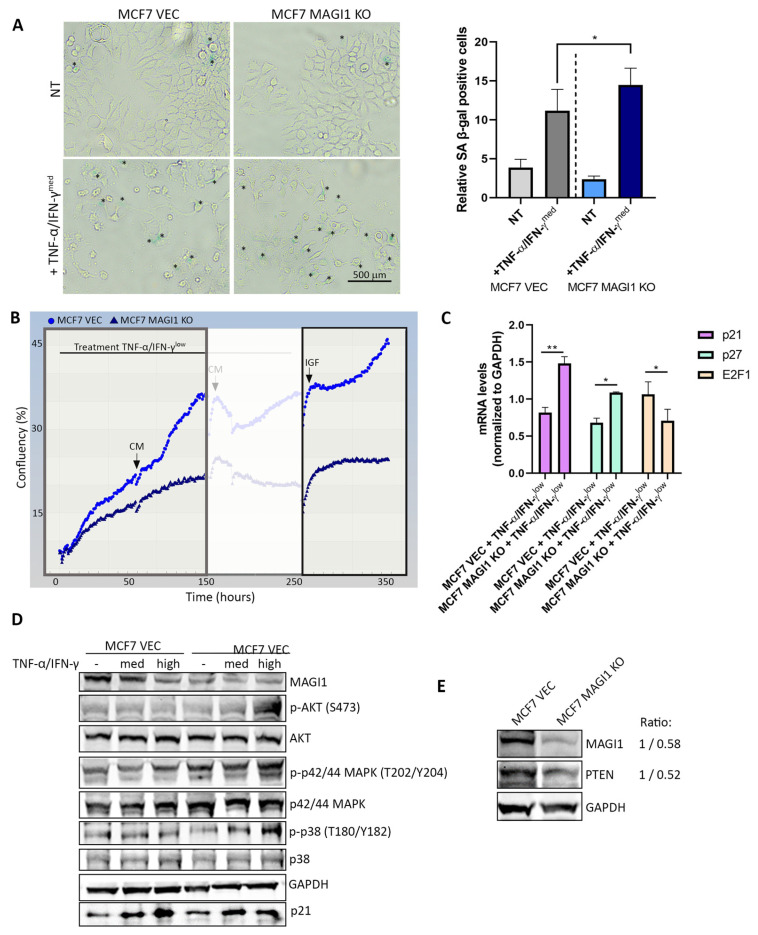
**MAGI1 loss in MCF7 cells promotes senescence and induces AKT and MAPK signaling in response to TNF-α/IFN-γ.** (**A**) SA β-gal-positive (blue) stained cells after 48 h treatment with an intermediate concentration of TNF-α/IFN-γ. Representative images are shown. Blue-stained cells are marked with *. Scale bar 500 μm. The percentage of SA-β-gal positive stained cells is shown on the right (n = 3 independent experiments; * *p* ≤ 0.05). (**B**) Real-time growth curves show that MCF7 MAGI1 KO cells (dark blue) proliferate slower in the presence of TNF-α/IFN-γ than MCF7 VEC cells (light blue), as shown in the left part of the graph (grey rectangle). TNF-α/IFN-γ-treated MCF7 MAGI1 KO cells do not respond to insulin growth factor (IGF) stimulation in contrast to MCF7 VEC cells (right part of the graph, black rectangle). (**C**) After 7 days of treatment with a low concentration of TNF-α/IFN-γ, the mRNA levels of the CDK inhibitors *p21* and *p27* are significantly higher and the levels of *E2F1* are lower in MCF7 MAGI1 KO cells compared with MCF7 VEC cells (n = 3 independent experiments; * *p* ≤ 0.05, ** *p* ≤ 0.01). (**D**) Western blot analyses of different proteins after 24 h of intermediate (med) and high concentrations of TNF-α/IFN-γ exposure. MCF7 MAGI1 KO cells show an increase in phospho-AKT (S473), phospho-p44/42 MAPK (T202/Y204), and phospho-p38 (T180/Y182) protein levels. GAPDH and p21 were used as loading and senescence induction controls, respectively. (**E**) PTEN protein levels are decreased in MCF7 MAGI1 KO cells when compared with MCF7 VEC cells. The densitometric ratio of MAGI1 and PTEN protein levels in MCF7 VEC/MCF7 MAGI1 KO is shown next to the blot.

Tumor cell senescence can be induced in vitro following combined exposure to TNF-α and IFN-γ, two cytokines that are endogenously produced by tumor-infiltrating Th1 helper lymphocytes [60]. Combined exposure to these cytokines also induces the senescence of MCF7 cells, as has been previously demonstrated [61]. For our study, we used high (20/33 ng/μL), intermediate (10/15 ng/μL), and low (0.5/0.75 ng/μL) TNF-α and IFN-γ concentrations for different purposes (for more details, see the Materials and Methods section). The highest concentration (20/33 ng/μL) was tolerated for a maximum of 48 h before the cells started to show signs of toxicity, and it was therefore used, together with the intermediate dose, for short treatments (typically up to 24 h). For long-term experiments, the lowest concentration of TNF-α and IFN-γ (0.5/0.75 ng/μL) was used. Due to the heterogeneous and dynamic nature of senescence, several markers are used to identify senescent cells [6]. The arrested cell cycle, dictated by the regulation of numerous key factors including cyclin-dependent kinase (CDK) inhibitors such as p21 (CDKN1A) [62] or p27 (CDKN1B) [9] together with senescence-associated β-galactosidase (SA-β-gal) staining are commonly used markers [63]. As observed in Figure 2A, the combination of TNF-α and IFN-γ resulted in a senescence-like morphology [6,60] that was apparent in both MCF7 VEC and MCF7 MAGI1 KO cells. Cells were more elongated and presented a more flattened morphology, losing their typical cobblestone appearance and adopting a fibroblast-like appereance, and they exhibited an increase in SA-β-gal activity, evidenced by the appearance of a blue stain. Quantification of the blue-stained cells showed that MCF7 MAGI1 KO cells had a significantly higher fraction of positive SA-β-gal-stained cells than MCF7 VEC cells (14.47% vs. 11.15%, *p* < 0.05). In order to evaluate the effect of TNF-α and IFN-γ at later timepoints, cells were exposed to low concentrations of TNF-α and IFN-γ over a period of 14 days and continuously monitored for growth using the IncuCyte^TM^ imaging system. As seen in Figure 2B (left part of the graph), MCF7 VEC cells that were simultaneously treated with TNF-α and IFN-γ proliferated more under these conditions when compared with MCF7 MAGI1 KO cells, and this difference became more pronounced at later timepoints (already after 72 h). When cells were subsequently stimulated with insulin growth factor (IGF) 10 days after the TNF-α/IFN-γ treatment started, MCF7 VEC cells resumed proliferation, indicating that they were not irreversibly arrested (Figure 2B, right part). In contrast, MCF7 MAGI1 KO cells did not resume proliferation in response to IGF and remained quiescent, which is consistent with an irreversible arrest or senescent phenotype.

The deeper the quiescence, the stronger and longer the growth signal must be to ‘revive’ the cells; deep quiescent cells have a higher E2F switching threshold than cells in shallow quiescence [64]. The E2F threshold represents the strength of the growth signal(s) required for a cell to exit quiescence and re-enter the cell cycle, and CDK inhibitors (e.g., p21, p27) raise this threshold. When we analyzed the mRNA levels of the different CDK inhibitors and *E2F1*, a transcription factor that induces quiescent cells to re-enter the S phase [65], we found that after 7 days of TNF-α and IFN-γ induction, the mRNA expression of *p21* and *p27* was significantly higher in MCF7 MAGI1 KO cells when compared with MCF7 VEC cells, whereas mRNA levels of *E2F1* were lower (Figure 2C). Therefore, MCF7 MAGI1 KO cells presented a higher E2F-activation threshold than MCF7 VEC cells, meaning that these cells were in a more ‘deep-quiescent/senescence’ phenotype; meanwhile, MCF7 VEC cells were in ‘shallow quiescence’.

A Western blot analysis of cells treated with high concentrations of TNF-α and IFN-γ for 24 h revealed a significant increase in the protein levels of phospho-AKT at Ser473 (S473), phospho-p42/44 MAPK (ERK1/2) at Thr202/Tyr204 (T202/Y204), and phospho-p38 MAPK at Thr180/Tyr182 (T180/Y182) in MCF7 MAGI1 KO cells compared with MCF7 VEC cells (Figure 2D, quantification of phosphorylated proteins in Figure S3A). Activation of AKT, MAPK (ERK1/2), and p38 has been shown to promote the survival of senescence cells in response to stress [66]. Furthermore, the loss of the tumor suppressor PTEN, the major negative regulator of the PI3K/AKT pathway, was shown to induce senescence in several cell types [67]. PTEN is a known binding partner of MAGI1 [68], and therefore, we analyzed the protein levels of PTEN in MCF7 MAGI1 KO cells. As shown in Figure 2E, MCF7 MAGI1 KO cells have lower PTEN protein levels than MCF7 VEC cells.

From these observations, we conclude that upon long-term exposure (≥7 days) to TNF-α and IFN-γ, MAGI1 low MCF7 cells enter a senescence-like state that is associated with an increased expression of *p21* and *p27*, decreased expression of *E2F1*, and increased phosphorylation (activation) of AKT, as well as p42/44 and p38 MAPK.

### 3.3. MCF7 MAGI1 KO Cells Fail to Activate DNA Repair Proteins, Accumulate DNA Damage, and Induce the PI3K/AKT Pathway after Exposure to Ionizing Radiation (IR)

**Figure 3:**
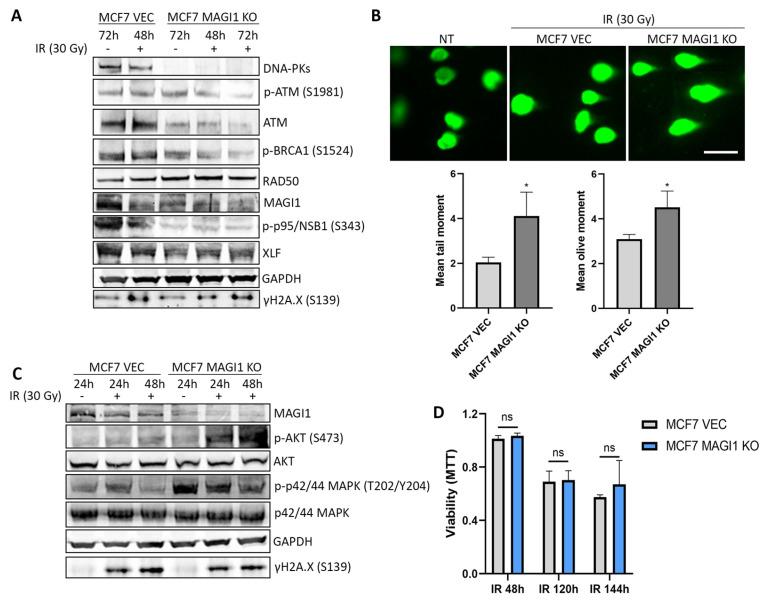
**MAGI1 loss impairs the DNA damage response and activates the AKT and MAPK signaling pathways in response to X-ray irradiation without affecting cell viability.** (**A**) Western blot analysis showing that relevant proteins implicated in DNA DSBs repair are downregulated in MCF7 MAGI1 KO cells in both non-treated and cells treated with ionizing radiation (IR 30 Gy) when compared with MCF7 VEC cells. GAPDH and γH2A.X were used as loading and DNA damage controls, respectively. (**B**) Comet assay images showing comet tails in MCF7 VEC and MCF7 MAGI1 KO cells after 30 Gy-IR. As a non-treated condition, a mix of 1:1 of MCF7 VEC and MCF7 MAGI1 KO cells was used. Scale bar 75 μm. Significant differences in the mean tail moment (left) and mean olive moment (right) between MCF7 VEC and MCF7 MAGI1 KO cells 48 h after 30 Gy-IR are shown (n = 2 independent experiments; * *p* ≤ 0.05). (**C**) Phospho-AKT (S473) and phospho-p42/44 MAPK (T202/Y204) protein levels are higher in MCF7 MAGI1 KO cells than MCF7 VEC cells after exposure to 30 Gy IR. (**D**) Cell viability measured using the MTT assay at 48, 120, and 144 h post 30 Gy-IR, normalized to NT (n = 3 independent experiments; ns indicates not significant *p* > 0.05).

Persistent or unresolved DNA damage [9] and a lack of DNA repair gene expression [69] can induce senescence. As the GSEA analyses revealed alterations in the DNA damage checkpoint gene set in MCF7 MAGI1 KO cells, we sought to investigate the DNA damage response in MCF7 cells exposed to agents causing double strand breaks (DSBs). DSBs are highly cytotoxic and, if repaired improperly, can cause oncogenic chromosome translocations [70]. We investigated the level of total and phosphorylated proteins that are crucially involved in the two main DDR pathways, HR and NHEJ, after inducing DNA damage by ionizing radiation (IR). A dose of 30 Gray (Gy) triggered a rapid phosphorylation of the histone variant H2AX, producing γH2AX in both MCF7 VEC and MCF7 MAGI1 KO cells after 48 h (Figure 3A). Phosphorylation of H2AX plays a key role as a sensor in DDR and is required for the assembly of DNA repair proteins at the sites containing damaged chromatin [71]. Upon sensing the DNA damage, a coordinated activation of DNA damage checkpoints as well as DNA repair proteins is required to arrest the cell cycle, thus allowing for repair [72]. DNA-PKs and ATM proteins mainly mediate the repair of DNA DSBs through the NHEJ and HR pathways, respectively [73]. After 48 h of exposure to X-ray irradiation with 30 Gy, different proteins belonging to the HR repair pathway such as phospho-ATM (S1981), phospho-BRCA1 (S1524), or phospho-p95/NSB1 (S343), as well as DNA-Pks and XLF, both of which are involved in the NHEJ repair pathway, were active in MCF7 VEC cells. In MCF7 MAGI1 KO cells, the basal levels of DNA-PKs, ATM, and phospho-p95/NSB1 (S343) were lower compared with MCF7 VEC cells, and there was no activation of any of these DNA repair proteins after 48 h and 72 h post-irradiation (Figure 3A).

To test whether deficient DNA repair protein expression translated into deficient DNA repair, we used the comet assay, a sensitive method that is widely used to demonstrate DNA DSBs and fragmentation in individual cells [74,75]. A quantitative analysis of the comets showed significantly increased comet tail formation following X-ray exposure in both MCF7 VEC and MCF7 MAGI1 KO cells (Figure 3B), indicating unrepaired DNA damage. MCF7 MAGI1 KO cells were more affected than MCF7 VEC cells as seen by a significant increase in both the tail and the olive moments, two parameters used to measure the extent of the DNA damage. The tail moment combines the tail length and tail intensity into one single value [76], while the olive moment measures the heterogeneity within a cell population [77].

Interestingly, exposure to 30 Gy-IR resulted in an increase in phospho-AKT (S473) protein levels in MCF7 MAGI1 KO cells compared with MCF7 VEC cells (Figure 3C, quantification of phosphorylated proteins shown in Figure S3B). MAGI1-low MCF7 cells have constitutively high levels of phospho-p42/44 MAPK (T202/Y204) when compared with MCF7 VEC cells; irradiation does not increase these levels, but rather, they are maintained after 24 and 48 h. High levels of phospho-AKT can inhibit the HR repair pathway by suppressing the formation of BRCA1 and RAD51 foci after exposure to IR in BC [78]. Consistently, PTEN-deficient cells also fail to resect DSBs efficiently after IR and show a greatly diminished proficiency of the HR pathway [79]. As in the presence of a defective DNA repair system, DNA damage often correlates with apoptosis [80], we investigated whether MCF7 MAGI1 KO cells had decreased cell viability post-irradiation. Cells exposed to 30 Gy-IR were barely affected after 48 h (Figure 3D). After 120 h post-irradiation, however, viability was similarly decreased by 40% in both cell lines. At a later timepoint (144 h), viability decreased by approximately 50%; however, there were no significant differences between MCF7 VEC and MCF7 MAGI1 KO cells.

From these experiments, we conclude that MAGI1 low MCF7 cells have a constitutively lower level of proteins that are critically involved in DNA repair, and these cells fail to activate them in response to exposure to DNA damaging X-ray irradiation. In addition, cells accumulate more DNA DSBs, as demonstrated by the comet assay. These observations suggest that MAGI1-low MCF7 cells may have a deficient DNA damage response and repair activity.

### 3.4. Transcriptome Analyses of Human Patients Shows a Correlation between Low MAGI1 Levels and Increased Tumor Mutational Burden (TMB), Homologous Recombination Deficiency (HRD), and AKT/MAPK Signaling

HRD refers to the inability to efficiently repair DNA DSBs using the HR repair pathway [81]. In addition, inactivation or defects in DNA repair genes as well as cells with a reduced ability to undergo apoptosis in response to DNA damage tend to accumulate mutations [82]. As MAGI1-low MCF7 cells have a lower level of proteins that are critically involved in DNA repair and because these cells fail to activate them in response to DNA damage, we hypothesize that the loss of MAGI1 would correlate with a higher HRD and TMB in patients. When looking at patients with ER^+^HER2^−^ BCs from the ICGC dataset, we indeed observed a negative correlation between *MAGI1* mRNA expression levels and the HRD scores (rho = −0.22, *p*-value < 0.001; Figure S4A) along with the TMB scores (rho = −0.24, *p*-value < 0.001; Figure S4B), which confirmed our hypothesis. By using patients with ER^+^HER2^−^ BCs from the TCGA dataset, we could investigate the transcriptomic landscape according to the *MAGI1* mRNA expression levels. At the transcriptomic level, MAGI1 expression was positively correlated with the ESR1 signature and negatively correlated with proliferation and AKT/MAPK pathways, as indicated by the negative associations observed in multiple gene signatures (Figure S4C).

### 3.5. MCF7 MAG1 KO Cells Are More Sensitive to the Combination of Cisplatin and Pharmacological Inhibition of PARP1

**Figure 4:**
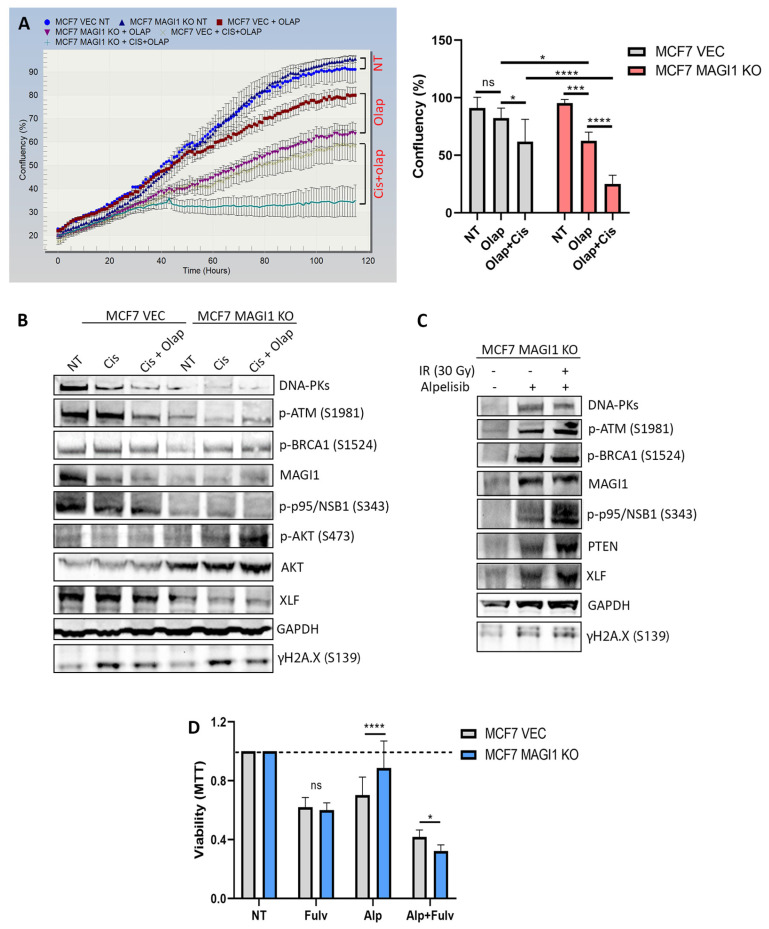
**MAGI1-low MCF7 cells are sensitive to PARP inhibition and combined alpelisib/fulvestrant treatment and reactivate the DNA damage response proteins upon PI3K inhibition.** (**A**) MCF7 MAGI1 KO cells are more sensitive to olaparib and to the combination of olaparib and cisplatin than MCF7 VEC cells, as observed in the real-time growth curves (confluency) measured up to 120 h (left). The analysis of the end timepoint confluency is represented in the graph on the right; ns indicates not significant *p* > 0.05, * *p* ≤ 0.05, *** *p* ≤ 0.001, **** *p* ≤ 0.0001. (**B**) Western blot showing the main proteins involved in DNA repair. Levels of DNA-PKs, phospho-ATM (S1981), phospho-BRCA1 (S1524), phospho-p95/NSB1 (S343), and XLF are downregulated in MCF7 MAGI1 KO cells when compared with MCF7 VEC cells in both non-treated cells and cells treated with olaparib, cisplatin, or the combination of olaparib and cisplatin. Phospho-AKT (S473) is active in MCF7 MAGI1 KO cells after treatment with olaparib and cisplatin when compared with MCF7 VEC cells. GAPDH and γH2A.X were used as loading and DNA damage controls, respectively. (**C**) The main proteins implicated in DNA repair (DNA-PKs, phospho-ATM (S1981), ATM, phospho-p95/NSB1 (S343), and XLF) are upregulated after treatment with the PI3K inhibitor alpelisib in MCF7 MAGI1 KO cells. (**D**) Viability (MTT) measured after treatment of alpelisib and co-treatment of alpelisib and fulvestrant in MCF7 VEC and MCF7 MAGI1 KO cells (n = 3 independent experiments; ns indicates not significant *p* > 0.05, * *p* ≤ 0.05, **** *p* ≤ 0.0001).

Defects in DNA repair increases the susceptibility of the cells to DNA-damaging agents [83]. The use of Poly-[ADP-ribose]-polymerase-1 (PARP1) inhibitors results in synergistic antitumor effects when combined with DNA-damaging agents such as cisplatin or X-ray irradiation [84]. Because MCF7 MAGI1 KO cells showed defects in their DNA repair machinery, we tested whether olaparib, a PARP1 inhibitor used in patients to treat certain subtypes of breast and ovarian cancers, would render MCF7 MAGI1 KO cells more sensitive to cisplatin. Cisplatin causes DNA damage by interfering with DNA repair mechanisms, resulting in the induction of cancer cell apoptosis [85]. Previous studies evaluating the effects of cisplatin on the MCF7 cell line have reported that this line is relatively resistant compared with other BC cell lines [86]. A concentration of 60 μM of cisplatin was required to reduce the viability of MCF7 VEC and MCF7 MAGI1 KO cells by 50% (Figure S5A), which is consistent with previous reports [86], and no significant differences were observed regarding the cell proliferation between both cell lines (Figure S5B). The use of olaparib alone decreased the proliferation of MCF7 VEC and MCF7 MAGI1 KO cells (Figure 4A), albeit the decrease was more pronounced in MCF7 MAGI1 KO cells. When cisplatin (40 μM) was combined with olaparib, the decrease in cell proliferation and viability was more evident in MCF7 MAGI1 KO cells than in MCF7 VEC cells (Figure 4A and Figure S5A). To test whether the increased sensitivity of MCF7 MAGI1 KO to combined cisplatin and olaparib treatment was due to a synergistic or additive effect, we transformed the cell confluency values of cisplatin, olaparib, and cisplatin + olaparib treatments into inhibition values (efficacy) upon adjustment to no treatment values (Figure S5C). The obtained values were analyzed using the response additivity approach, also known as the linear interaction effect [87]. The results indicated that the increased sensitivity of MCF7 MAGI1 KO to combined cisplatin and olaparib treatment was due to an additive effect (cisplatin, 39% + olaparib, 34.31% = 73.31% ≈ combined, 73.72%). The same analysis applied to MCF7 VEC cells treated with cisplatin, olaparib, and cisplatin + olaparib revealed no additive effect but rather a highest single agent effect (cisplatin, 35.12% ≈ combined, 32.12%). 

When we analyzed the different DNA repair proteins belonging to the two main repair pathways (HR and NHEJ), we observed that after treatment with cisplatin alone or after the combination of cisplatin (40 μM) with olaparib, MCF7 MAGI1 KO cells failed to activate the DNA damage response (Figure 4B); the protein levels of DNA-PKs, phospho-ATM (S1981), phospho-BRCA1 (S1524), phospho-p95/NSB1 (S343), and XLF remained low when compared with the levels of these proteins in MCF7 VEC cells under the same conditions. We again observed an increase in the levels of active phospho-AKT (S473) in MCF7 MAGI1 KO cells after co-treatment with cisplatin and olaparib. Previous studies have described that PARP inhibitors have favorable anti-tumor effects on breast and ovarian cancers with defective repair pathways [88].

Our observations indicate that MCF7 MAGI1 KO cells are more sensitive to PARP1 inhibition alone or in combination with cisplatin, implying that patients with defective DNA repair pathways due to low MAGI1 levels could potentially benefit from this treatment approach.

### 3.6. The PI3K Inhibitor Alpelisib Sensitizes MCF7 MAGI1 KO Cells to Fulvestrant

AKT can be activated in response to genotoxic insults induced by anticancer therapies [89], due to activating mutations in PI3K (*PI3KCA*), or due to the loss of the tumor suppressor PTEN (reviewed in [90]). In view of the potent oncogenic activity of the PI3K/AKT signaling pathway, substantial efforts have been made to target this pathway for therapeutic purposes. Because we have persistently observed an activation of AKT in MCF7 MAGI1 KO cells under different types of stress (i.e., after TNF-α/IFN-γ-induced senescence or after IR and cisplatin/olaparib treatments), we decided to evaluate the effect of blocking this pathway with the PI3Kα-selective inhibitor alpelisib [91], on the different proteins involved in DNA repair in MCF7 MAGI1 KO cells. As shown in Figure 4C, treatment with alpelisib (alone or in combination with 30 Gy-IR) triggered the activation of different proteins involved in DNA repair from the two main pathways, i.e., HR and NHEJ. Surprisingly, alpelisib also induced an increase in the protein levels of MAGI1 and PTEN. Treatment with the AKT inhibitor MK-2206 showed similar results (Figure S6A). Therefore, we conclude that AKT is impairing DNA repair events in MCF7 MAGI1 KO cells.

A role of AKT in impairing DNA repair was already reported, and similarly, inhibition of AKT restored the DNA damage response in irradiated cells [92]. Next, we measured the viability of the cells after alpelisib treatment alone and in combination with fulvestrant, an ER antagonist. As shown in Figure 4D, MCF7 MAGI1 KO cells were significantly more resistant to alpelisib than MCF7 VEC cells. This is most likely due to residual levels of AKT signaling still being present or the possibility of AKT activation mechanisms that can occur despite an effective inhibition of PI3Kα [93]. AKT positively regulates mTOR, and similarly, the mTOR complex also activates AKT by phosphorylation, creating a network of regulatory loops [94].

As shown in Figure 1A, the transcriptome analyses revealed that the mTOR signaling pathway was enriched in MCF7 MAGI1 KO cells; thus, we investigated the levels of phospho-mTOR (S2448). Indeed, we found that phospho-mTOR (S2448) levels were high in non-treated MCF7 MAGI1 KO cells and further increased following treatment with alpelisib (Figure S6B). In contrast, levels of phospho-mTOR (S2448) were lower in MCF7 VEC cells treated with alpelisib when compared with non-treated cells. The observed increased resistance of MCF7 MAGI1 KO cells towards alpelisib was abolished when the cells were simultaneously co-treated with fulvestrant. While MCF7 VEC cells showed a reduction in viability by 60% after this combination treatment, the decrease in MCF7 MAGI1 KO cells viability was around 70% (Figure 4D).

From these observations, we conclude that MAGI1 low cells respond to PI3K inhibition by restoring the DNA repair proteins and are more sensitive to the combination of PI3K inhibition with fulvestrant.

### 3.7. Pharmacological and Genomic Evidence for Transcriptional Regulation of MAGI1 Expression by HDACs

**Figure 5:**
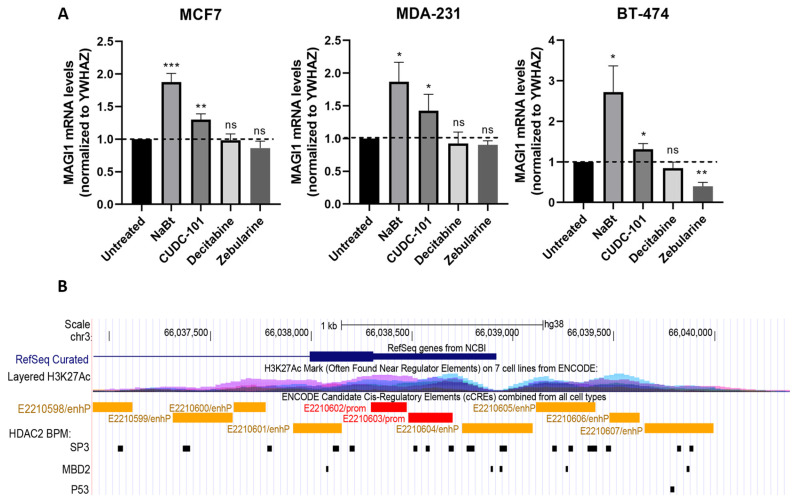
(**A**) **Pharmacological and genomic evidence for the transcriptional regulation of MAGI1 expression by HDACs.** *MAGI1* mRNA levels are increased after treatment with the HDAC inhibitors NaBt and CUDC-101 in MCF7, MDA-231, and BT-474 cells. No increase in *MAGI1* mRNA levels are observed after treatment with the DNMT inhibitors decitabine and zebularine in any of the tested lines (n = 3 independent experiments; ns indicates not significant *p* > 0.05, * *p* ≤ 0.05, ** *p* ≤ 0.01, *** *p* ≤ 0.001). (**B**) A computational search for predicted histones acetylation sites (H3K27Ac) and DNA binding sites of HDAC2-associated transcription factors near the promoter region of MAGI1 (RefSeq Curated). BPM: Binding-partners motifs.

MAGI1 expression is decreased in some inflammatory diseases and in several cancers, including hepatocellular carcinoma, colorectal, cervical, brain, and gastric cancers (reviewed in [17]). Mechanisms such as mechanical stress or inflammation regulate MAGI1 expression; however, epigenetic mechanisms, including DNA methylation and/or the deacetylation of histones, which may control the sustained up- or down-regulation of MAGI1 expression, have not been described yet. DNA methylation and histone deacetylation can repress gene transcription, and the use of either DNA methyltransferase inhibitors (DNMTis) or HDAC inhibitors (HDACis) can reactivate epigenetically silenced genes [95]. To test whether these epigenetic mechanisms could play an active role in suppressing MAGI1 expression, we exposed MCF7 wild-type (wt) cells to DNMTis and HDACis and evaluated *MAGI1* mRNA expression levels. Two different HDACis, namely sodium butyrate and CUDC-101, were used. Sodium butyrate (NaBt), a naturally occurring short-chain fatty acid that is a byproduct of carbohydrate metabolism in the gut, is one of the most widely studied HDACis [96]. When MCF7 wt cells were exposed to a concentration of 2.5 mM of NaBt, we observed a 1.8-fold increase in the mRNA levels of *MAGI1* (Figure 5A). To test whether the induction of *MAGI1* mRNA expression by NaBt MCF7 was cell-specific or not, we tested the cell lines BT-474 (ER^+^HER2^+^) and MDA-231 (TNBC). We found that NaBt treatment also increased *MAGI1* mRNA levels in these cell lines. We used another HDAC inhibitor to confirm our results. CUDC-101 is a potent HDAC inhibitor that synergistically inhibits EGFR/HER2 kinases and attenuates multiple compensatory pathways, such as AKT; it also suppresses the progression of a broad range of tumor types in both in vitro and in vivo xenograft models [97]. A concentration of 0.5 μM was sufficient to increase *MAGI1* mRNA levels in MCF7 cells. This increase was also observed in BT-474 and MDA-231 cells that were treated with CUDC-101. Regarding DNMTis, we examined the effect on *MAGI1* mRNA expression after treatments with decitabine and zebularine, two compounds that, when used at low doses, reduce genomic DNA methylation [98]. In this case, we observed that none of these inhibitors increased *MAGI1* mRNA levels in MCF7 cells at any tested timepoint (24 and 48 h). Similarly, in MDA-231 and BT-474 cells, the use of these inhibitors did not increase *MAGI1* mRNA levels.

In BT-474 cells, we observed that zebularine actually led to a significant decrease in *MAGI1* mRNA levels. Zebularine, besides acting as a DNMT inhibitor, induces S phase arrest and changes in the expression of cell cycle regulatory proteins at low doses and induces apoptosis at high doses in MCF7 and MDA-231 cells [99]. However, the cytotoxicity of this drug or its effect on different proteins has never been evaluated in BT-474 cells.

HDAC2 regulates gene expression via the deacetylation of the lysine residues in the N-terminus of core histones [100]. HDAC2 forms distinct transcriptional repressor complexes which, in turn, mediate chromatin remodeling. HDAC2 itself has not been described to bind to DNA directly, but the different HDAC2-associated complexes include DNA binding proteins such as SP3, MBD2, P53, and YY1 [101,102,103,104,105]. Therefore, to further characterize potential mechanisms of the transcriptional regulation of MAGI1 by HDAC2, we computationally searched for binding sites of HDAC2-associated transcription factors near the promoter region of MAGI1. We identified 46 binding sites for SP3, 7 sites for MBD2, and 2 sites for P53 in a 4 kb region surrounding the transcriptional start site of MAGI1 (Figure 5B). Notably, the majority of the binding sites identified overlapped with H3K27Ac-enriched regions obtained from H3K27Ac ChIP-seq data from human cell lines, as well as with candidate cis-regulatory elements identified by the ENCODE consortium [106,107]. These observations suggest that HDAC2 may regulate MAGI1 transcription through transcriptional complexes containing SP3 and/or MBD2. To further validate the predicted role of HDAC2 in regulating MAGI1 expression, we tested the effect of a specific HDAC2 inhibitor on MCF7 cells. We found a consistent upregulation in *MAGI1* mRNA expression levels after 24 h of treatment (Figure S7).

From these data, we conclude that histone deacetylation, but not DNA methylation, downregulates MAGI1 expression levels in the tested cell lines. Moreover, MAGI1 expression is regulated by HDACs, as observed by the transcriptional analysis of its promoter region.

## 4. Discussion

Tumor suppressor genes are generally involved in mediating senescence and in preventing DNA damage and/or promoting DNA repair, and their loss results in the inititation and progression of cancer [108,109]. In this study, we have demonstrated an unreported role of MAGI1 in protecting cells from deep quiescence/senescence as well as DNA damage. As deep quiescent cells share similar features and similar gene expression to senescent cells [110], the difference between them is sometimes indistinguishable. Here, we show that TNF-α- and IFN-γ-treated MCF7 VEC cells are able to re-enter the cell cyle upon exposure to a growth-stimulation signal, while MCF7 MAGI1 KO cells do not react and show a higher E2F activation threshold, indicating that MCF7 MAGI1 KO cells are either in a deep quiescent state or are fully senescent. Strikingly, MCF7 MAGI1 KO cells have lower levels of PTEN compared with MCF7 VEC cells, and both AKT and MAPK signaling pathways are active after senescence induction. This correlates with previous findings: the sustained hyperactivation of the PI3K/AKT/mTORC1 pathway results in cellular senescence [111,112] and the activation of MAPK pathways (mainly p38 and ERK1/2) also drives the senescence phenotype by exerting a direct control over the main senescence traits, i.e., cell survival, cell cycle arrest, and the senescence-associated secretory phenotype (SASP) [113]. Notably, the loss of PTEN can trigger senescence through a p53-dependent pathway called PTEN loss-induced cellular senescence (PICS), with mTOR being a key molecule involved in acting upstream and downstream of PI3K/AKT [114]. Cells can use senescence as an adaptive pathway to resist therapy, restart proliferation, and become more aggressive at a given timepoint [115]. Therefore, there is growing interest towards targeting these ‘dormant’ cells directly through the use of senolytic drugs or indirectly by targeting their survival mechanisms (reviewed in [116,117]). Previous studies on MAGI1 have shown that its downregulation in ER^+^HER2^−^ BC cells generates a more aggressive phenotype [21] and that silencing MAGI1 in colorectal cancer cells accelerates primary tumor growth and promotes metastasis [20]. The transcriptomic analyses performed in our study revealed that estrogen signaling, MYC, E2F targets, and the G2/M checkpoint gene sets were affected in MCF7 MAGI1 KO cells. The G2/M checkpoint and E2F transcription factors play critical roles in the cell cycle. It was recently reported that the G2/M checkpoint was associated with metastasis and poor survival in ER^+^HER2^−^ BC patients [118], while the E2F pathway was associated with aggressiveness and genomic aberrations [119]. Regarding MYC, it is a key regulator of cell growth, proliferation, and apoptosis, and its dysregulation contributes to BC development and progression, resistance to adjuvant therapy, and it is also associated with poor outcomes [120].

Loss of MAGI1 expression in ER^+^ BC patients also correlates with resistance to endocrine therapy and a worse outcome [21]. Activation of alternative growth pathways and/or cell survival mechanisms can lead to estrogen-independence and endocrine resistance [4]. Senescence and dysregulation of signaling pathways such as the PI3K/AKT and MAPK pathways [121] favor the development of tumor resistance. Other factors have been described, such as the existence of clonal subpopulations that continuously arise during treatment [122], which harbor defects in DNA repair mechanisms and contribute to a high somatic mutation load. In fact, it has been reported that defects in DNA repair pathways occur in ~40% of endocrine treatment-resistant ER^+^ BC patients [123]. Ionizing radiation and chemotherapeutic agents cause DNA damage [124]. We have seen in our study that MAGI1 loss impairs a proper DNA damage response, which is shown by an increase in the tail and olive moments following IR in the comet assay when compared with MCF7 VEC cells, indicating that DNA repair is, in fact, deficient in these cells. In line with these findings, cells with low MAGI1 levels showed a lack of activation of the main proteins that are involved in DNA repair pathways after exposure to IR or after cisplatin/olaparib treatments. Moreover, these cells were more sensitive to PARP1 inhibition. If DNA damage is left unresolved, there is a high risk of increased mutations and the possibility that damaged cells may become senescent and persist indefinitely [66]. In view of this, PARP1 inhibitors are being investigated for the treatment of earlier stages of BC in patients with somatic *BRCA* mutations but also in patients with mutations in other DNA damage repair genes [125]. An analysis of human BC patients’ gene expression data revealed that patients with low MAGI1 levels have higher TMB and HRD scores. A high HRD score has been shown to be predictive for clinical benefit when using PARP inhibitor therapy in some cancers, and the HRD status is now being incorporated as a predictive biomarker into prospective clinical trials [126]. The TMB score has also emerged as a useful biomarker for the evaluation of immunotherapy effectiveness in several cancer types. More recently, TMB was described as a biomarker for predicting overall survival, with high TMB scores being correlated with a reduced survival rate in BC patients [127]. In addition, *MAGI1* mRNA levels negatively associate with MAPK and PI3K/AKT signaling, which is correlated with our observations in vitro. Furthermore, PTEN levels were found to be negatively correlated with MAGI1 expression levels in the transcriptome analyses of human patients. Another interesting observation in this study is that after IR and cisplatin/olaparib treatments, MCF7 MAGI1 KO cells have active AKT signaling, which is known to contribute to tumor progression and drug resistance [128]. The PI3K/PTEN/AKT pathway plays a role in the regulation of the G2/M checkpoint. Cells with activated AKT can evade both the p53-independent G2/M cell cycle checkpoint and the apoptosis induced by DNA damage, thus they continue proliferating while accumulating mutations, leading to increased genome instability and resistance to genotoxic anticancer therapies [92,129]. AKT has also been described as a direct participant in the DNA damage response and repair process [89]. Xu et al. showed that AKT suppressed DNA damage processing and that inhibiting AKT restored the DNA damage-induced recruitment of proteins involved in DNA repair pathways [92]. Likewise, in this study, we show that inhibition of PI3K/AKT with either alpelisib, an α-selective PI3K inhibitor, or the specific AKT inhibitor MK-2206 restores DNA repair proteins. Unfortunately, to date, PI3K inhibitors have not achieved their expected therapeutic efficacy in clinical trials, which reflects the complex biology of this pathway and the possible compensatory mechanisms and highlights the need for combination treatment strategies and better ways to select for responding patients [130,131]. However, combination of the PIK3α inhibitor alpelisib with fulvestrant prolongs progression-free survival among ER^+^HER2^−^ BC patients with mutated *PIK3CA* that have relapsed under endocrine therapy [132]. Moreover, inhibiting PI3K/AKT enhances the apoptosis caused by other drugs such as trastuzumab or tamoxifen [133]. In line with this, we have seen in our study that MCF7 MAGI1 KO cells are more sensitive against the combination of alpelisib and fulvestrant than MCF7 VEC cells. In addition, to evade the activation of feedback loops, the combination of dual inhibitors against PI3K and mTOR (reviewed in [134]), as well as the simultaneous inhibition of the MAPK together with PI3K/mTOR signaling pathways [135], have shown efficacy in selected tumor types. Whether or not this triple combination could benefit patients with low MAGI1 levels would require further investigation.

A recurring question concerns the mechanism of MAGI1 downregulation in a subset of ER^+^HER2^−^ cancers [21]. Several studies have found that different MAGI subfamily members in different cell types are regulated by mutations, gene rearrangements, and methylation (reviewed in [17]). The use of HDAC inhibitors can reactivate epigenetically silenced genes [95] and thus represent a promising strategy for cancer therapy, particularly in combination with cytotoxic agents and/or radiotherapy [136]. In this study, we observed that *MAGI1* mRNA levels are increased after cell treatment with different HDAC inhibitors, i.e., NaBt, CUCD-101, and the HDAC2-specific inhibitor santacruzmate A. HDAC inhibitors similarly upregulate other tight junctions-associated proteins such as cingulin, ZO-1, ZO-2, or occludin [137]. A genomic analysis of the promoter region of MAGI1 provided supportive evidence for a regulation of MAGI1 expression by HDACs. Whether or not patients with low MAGI1 levels could potentially benefit from HDAC inhibitors in the clinic will require further evaluations. MAGI1 expression has also been shown to negatively correlate with inflammation in patients [21]. Senescent cells secrete a set of proteins (e.g., inflammatory cytokines, chemokines) known as the SASP, which influences both non-senescent normal cells and cancer cells in the tumor microenvironment and enhances tumor initiation [138]. Whether MAGI1 loss plays an active role by enforcing the senescence phenotype of the cells and consequently increasing inflammation, and if this in turn negatively regulates MAGI1 levels, is still an intriguing, open question.

## 5. Conclusions

**Figure 6:**
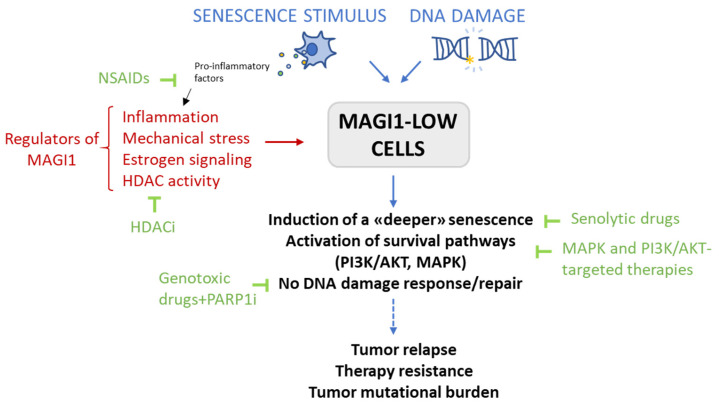
**Summary of cellular outcomes in cells with low MAGI1 levels after senescence induction and/or exposure to DNA damage, known regulators of MAGI1, and therapeutic approaches that could be potentially beneficial for ER^+^ BC patients with low MAGI1 levels.** Upon exposure to senescence stimuli and/or DNA-damaging agents, MCF7 MAGI1 KO cells show a deeper level of quiescence/senescence, activation of the AKT and MAPK survival signaling pathways, and an improper DNA damage response/repair, which would eventually lead to tumor relapse, therapy resistance, and a higher tumor mutational burden. MAGI1 has been shown to be regulated by inflammation, mechanical stress, estrogen signaling and, as shown here, HDAC activity. Regarding therapeutic approaches, PARP1 inhibition sensitizes MCF7 MAGI1 KO cells to cisplatin. Non-steroidal anti-inflammatory drugs (NSAIDs) increase MAGI1 expression [20,21], and could be used to maintain high MAGI1 levels. Finally, it is important to test whether patients with low MAGI1 levels could potentially benefit from the use of senolytic drugs, the concomitant targeting of underlying pro-survival pathways such as the PI3K/AKT and/or the MAPK signaling pathways, or the use of HDAC inhibitors.

This is the first study, to the best of our knowledge, that demonstrates a role of MAGI1 in regulating senescence and the DNA damage response. Our results suggest that MAGI1 simultaneously modulates the PI3K/AKT and MAPK pathways, which are critical survival pathways that induce senescence. We have also shown that loss of MAGI1 impairs a proper DNA damage response mediated by an overactivation of the PI3K/AKT pathway: blocking AKT restores DNA repair and increases MAGI1 protein levels. Whether the crosstalk between PI3K/AKT and MAGI1 is direct or is indirectly mediated by other pathways, molecules (i.e., through PTEN), or by more complex mechanisms is unclear at this point, and this question requires further investigation. The fact that MAGI1 is epigenetically modulated by histone deacetylation adds more information to possible mechanisms that contribute to MAGI1 loss during BC progression. We have proved that MAGI1 loss sensitizes cells to the PARP1 inhibitor olaparib and to the combination of the PI3K/AKT inhibitor alpelisib plus fulvestrant, which are therapeutic approaches that exploit either the DNA repair vulnerability of the cells or their PI3K/AKT-dependency. Based on these results, it would be important to test whether patients with low MAGI1 levels could potentially benefit from the use of senolytic drugs or the concomitant targeting of underlying pro-survival pathways such as the PI3K/AKT and/or MAPK signaling pathways. Similarly, it would be important to test whether HDAC inhibitors may increase MAGI1 levels in MAGI1 low ER^+^ tumors and prevent escape from hormonal therapy. The main findings of this work and their therapeutic implications are summarized in Figure 6.

In conclusion, the tumor suppressor MAGI1 is emerging as an important molecule in modulating different activities in cancer cells. This study has revealed the pathways and events that are affected by MAGI1 loss in ER^+^ BC cells and may open new strategies to improve the management of ER^+^ BC patients with low MAGI1 levels.

## Data Availability

RNASeq data were deposited in the Gene Expression Omnibus (GEO) repository under the accession number GSE237984.

## Supplementary Materials

The following supporting information can be downloaded at https://www.mdpi.com/article/10.3390/cells12151929/s1: Figure S1: Representation of the MAGI1 structure with targeted CRISPR regions and analysis of MAGI1 expression levels. Figure S2: Oncogenic signatures modulated by MAGI1 loss. Figure S3: Densitometric analysis of phosphorylated proteins. Figure S4: Association between MAGI1 mRNA expression levels, HRD, and TMB scores, and gene expression signatures in patients with ER+HER2- BCs. Figure S5: Cell morphology and proliferation analysis of MCF7 VEC and MCF7 MAGI1 KO cells exposed to different drugs. Figure S6: Western blot analysis of multiple proteins after exposure to different drugs and/or IR. Figure S7: HDAC2 inhibition in MCF7 cells increase MAGI1 expression levels. Table S1: KD_effect_seqA_and_B. Table S2: KD_effect_seqA_seqB_common_DN_UP. Table S3: GO_BP_UP_DOWN.
