# Supplementary material for: MAGI1 Prevents Senescence and Promotes the DNA Damage Response in ER+ Breast Cancer
Source: Cells. 2023 Jul 25;12(15):1929. doi: 10.3390/cells12151929 (PMC10417439; doi:10.3390/cells12151929)
Supplement: Supplementary file 1 [file cells-12-01929-s001.zip › Suppl_figure.pdf]

Supplementary Figures

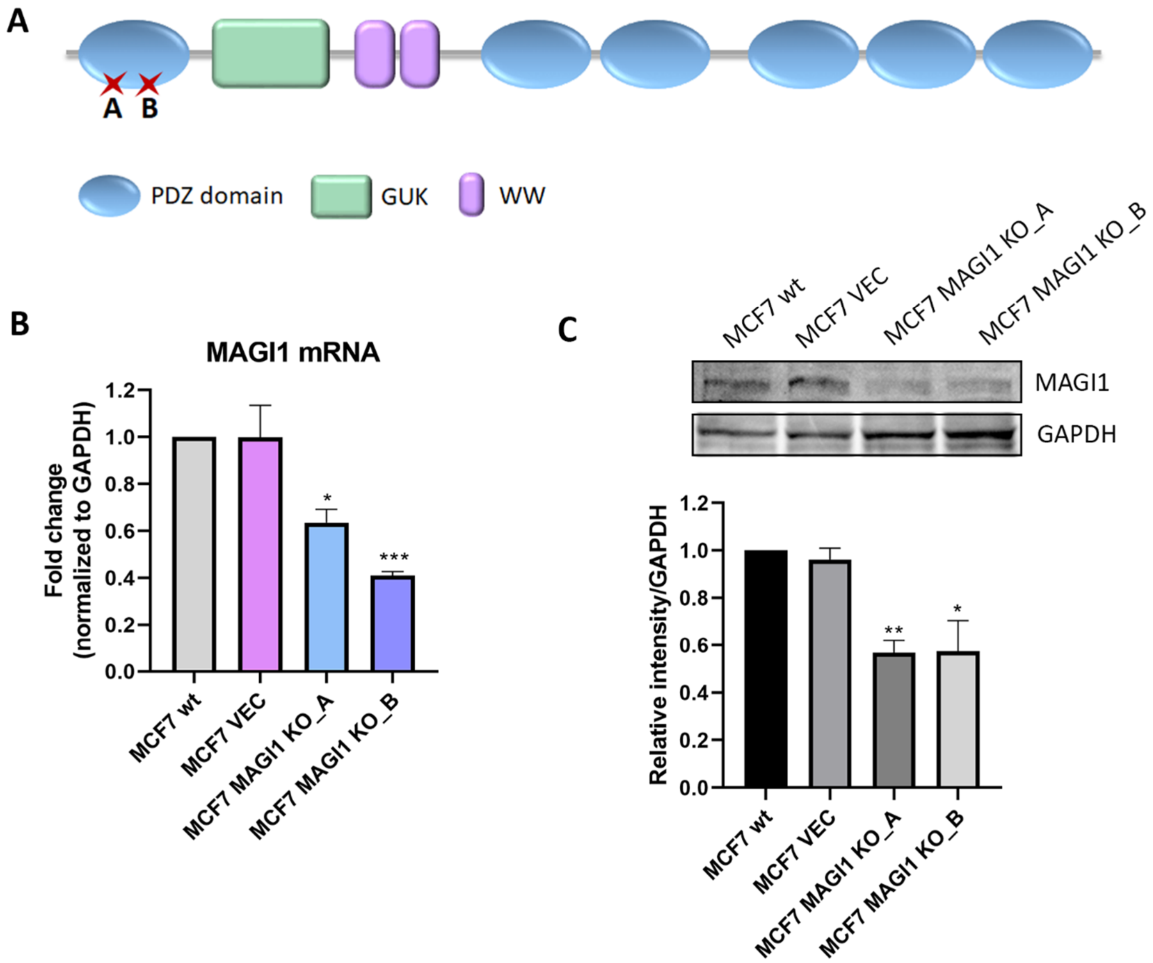

Figure S1. Representation of MAGI1 structure with targeted CRISPR regions and analysis of MAGI1 expression levels. (**A**) MAGI1 CRISPR-target sequences A and B target different regions in the PDZO domain of MAGI1. (**B**) Both MCF7 MAGI1 KO_A and MCF7 MAGI1 KO_B show a decrease of around 50-60% in *MAGI1* mRNA levels. (**C**) Western blot analysis of both MCF7 MAGI1 KO_A and MCF7 MAGI1 KO_B show a reduction in MAGI1 protein levels. Densitometric analysis of the western blot shows a reduction of 50% in MAGI1 protein levels. Normalized to GAPDH (* $p \leq 0.05$, ** $p \leq 0.01$, *** $p \leq 0.001$).

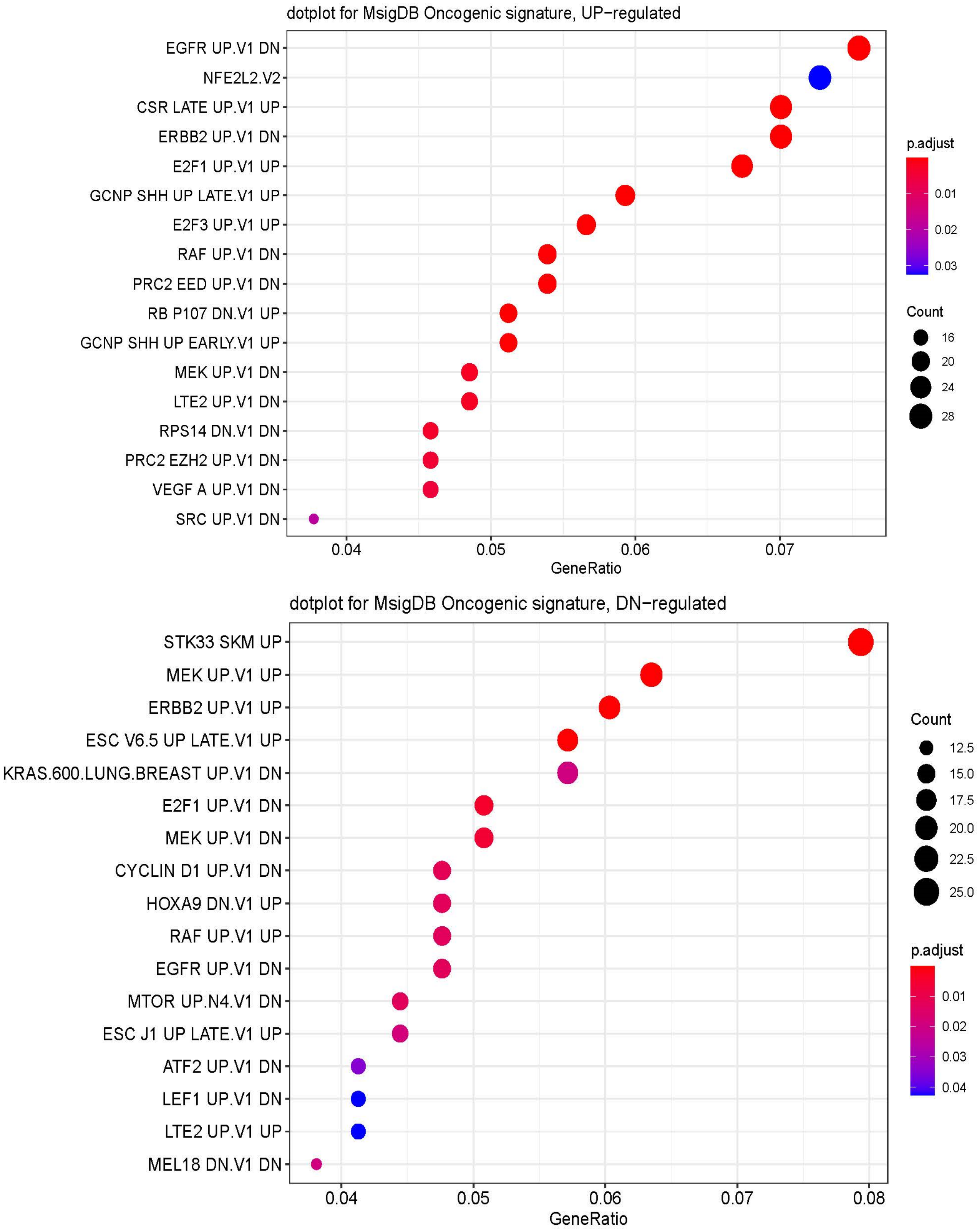

Figure S2. Oncogenic signatures modulated by MAGI1 loss. Dotplot for the Molecular Signatures Database (MSigDB) showing up-regulated (upper panel) and downregulated (lower panel) oncogenic signatures affected in MCF7 MAGI1 KO cells compared to MCF7 VEC cells.

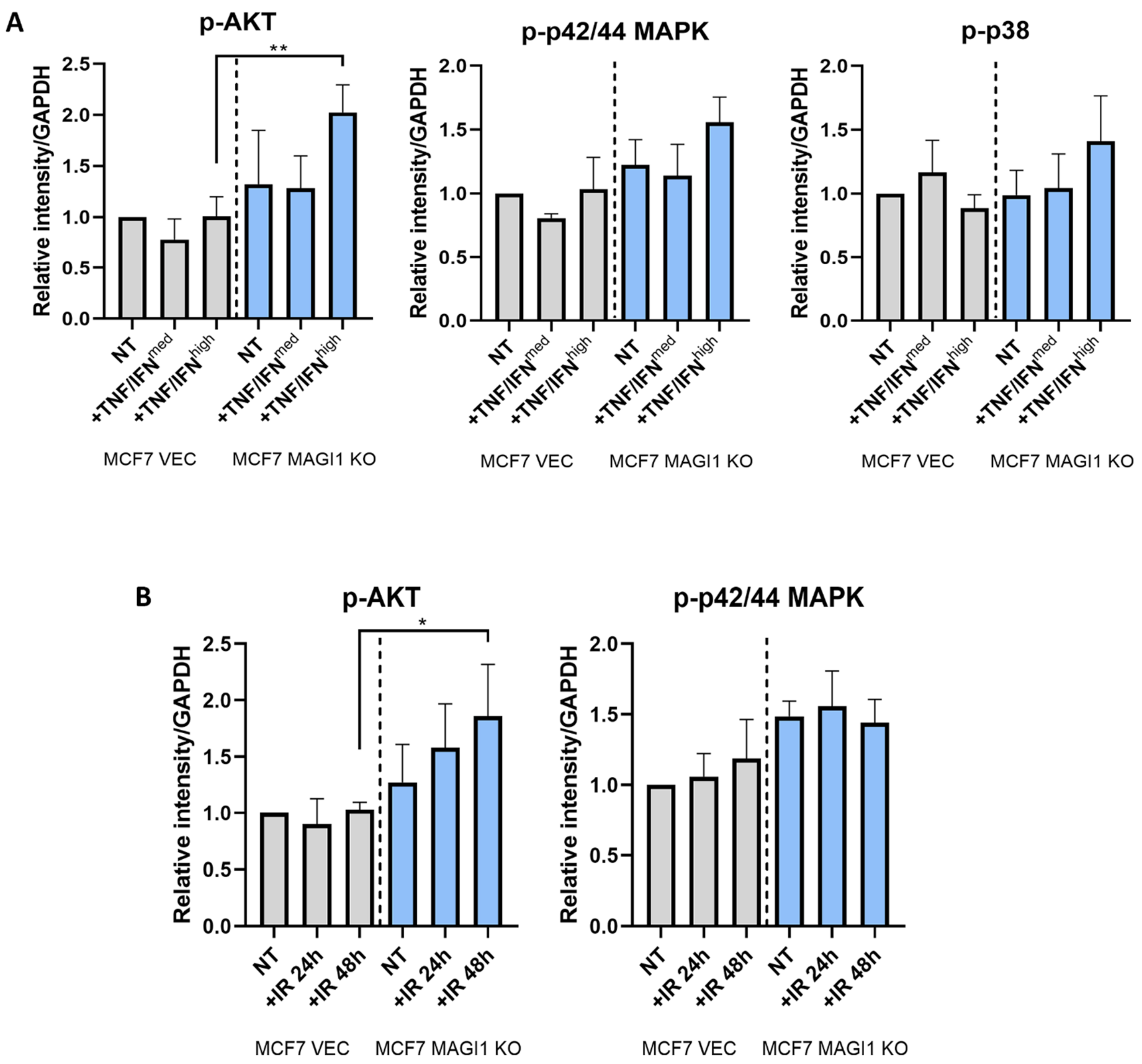

Figure S3. Densitometric analysis of phosphorylated proteins. Relative intensity of the phosphorylated proteins phospho-AKT (S473), phospho-p42/44 MAPK (T202/Y204) and phospho-p38 (T180/Y182) compared to MCF7 VEC NT measured by densitometric analysis of western blot and normalized to GAPDH (* $p \leq 0.05$, ** $p \leq 0.01$).

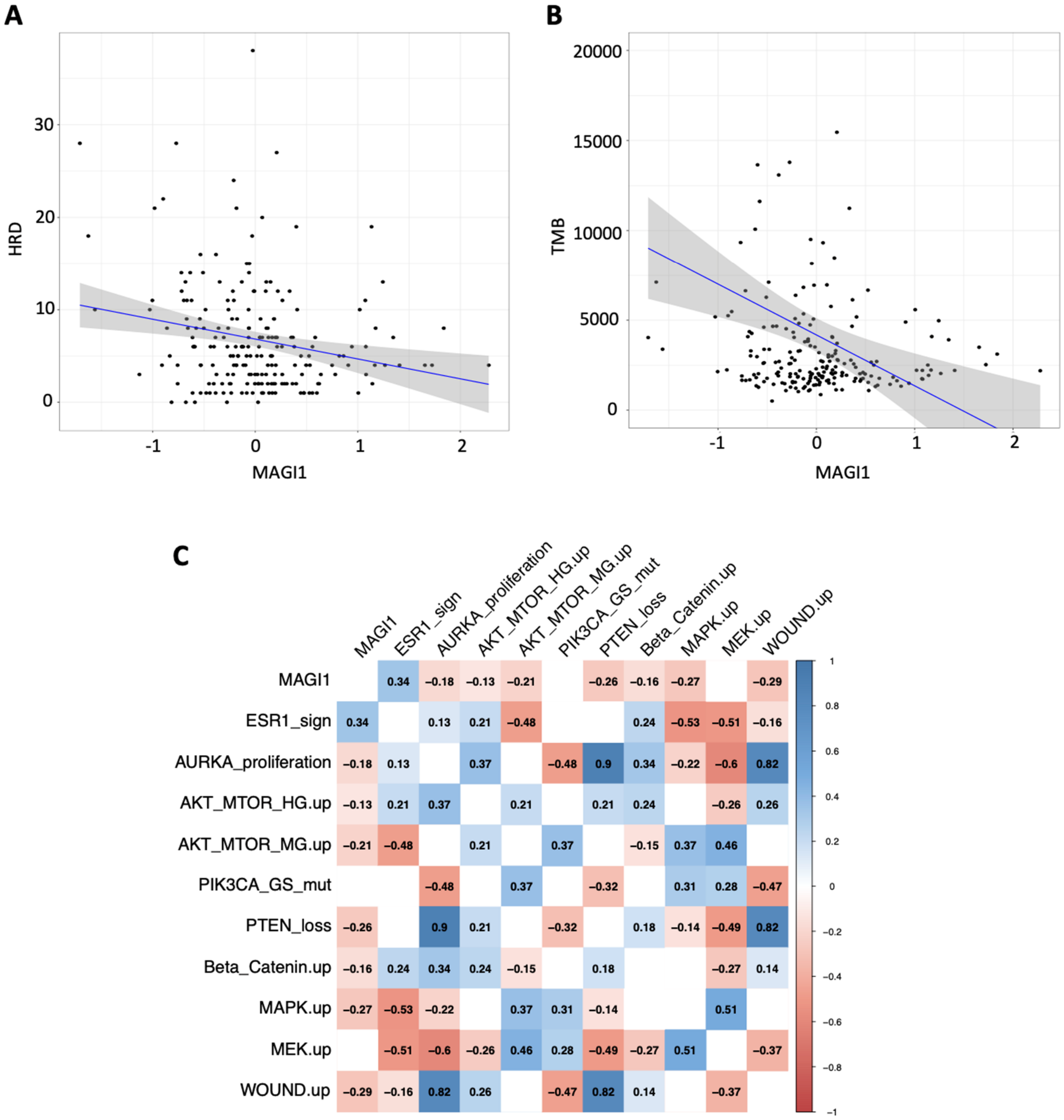

Figure S4. Association between *MAGI1* mRNA expression levels, HRD and TMB scores, and gene expression signatures in patients with $ER^{+}HER2^{-}$ BCs. Data were retrieved from the ICGC dataset, 206 and 207 patients were considered for the HRD (**A**) and the TMB (**B**), respectively. The blue line represents the linear regression together with the 95% confidence interval in grey. HRD spearman rho= -0.22, *p*-value < 0.001; TMB spearman rho= -0.24, *p*-value < 0.001. (**C**) *MAGI1* mRNA expression levels correlate positively with ESR1 signature and negatively with AKT and MAPK pathways signatures. Data was retrieved from the TCGA dataset and 290 patients were considered for the gene signature. Only significant associations are colored, positive in blue, negative in red. The spearman rho is reported in black.

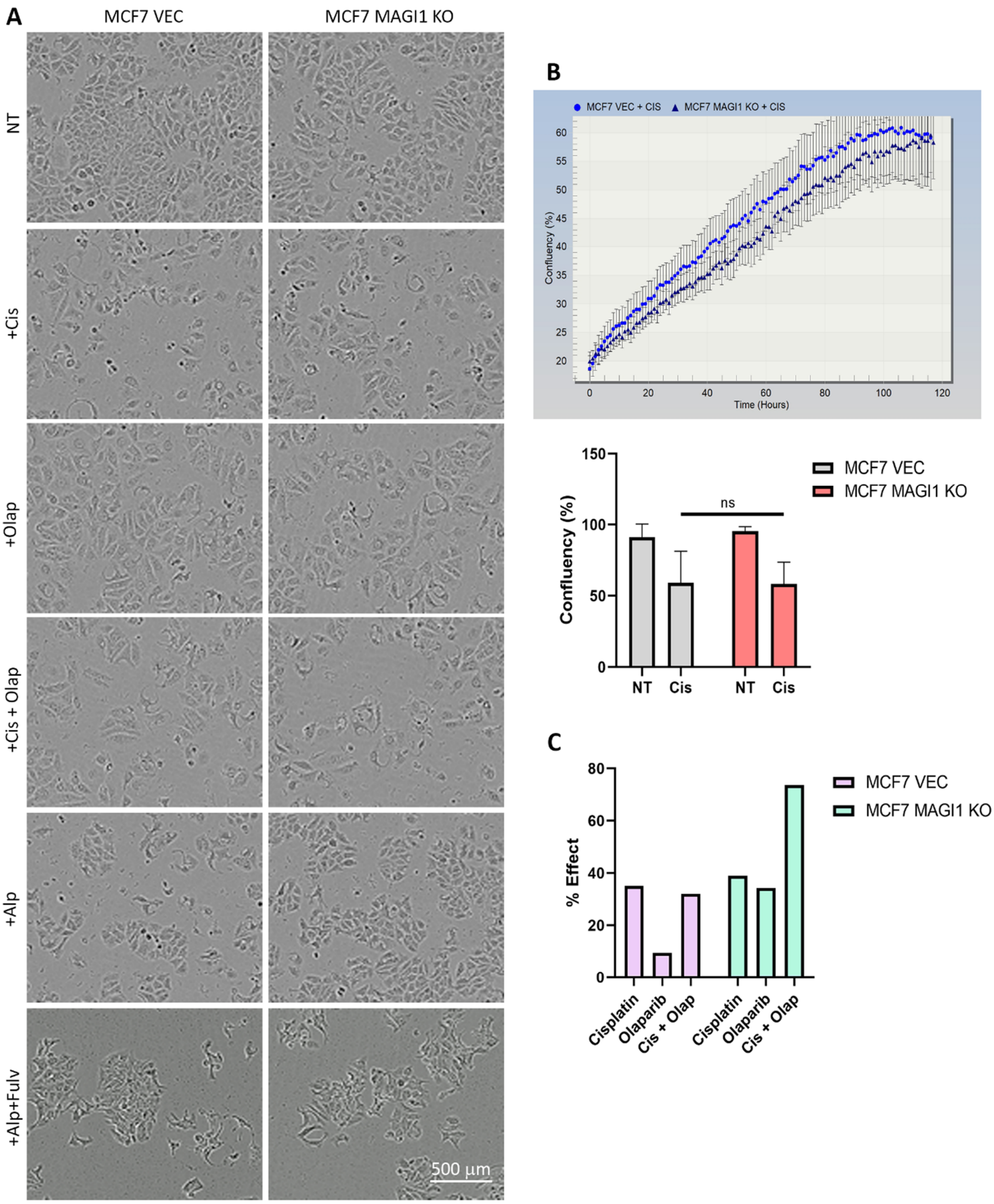

Figure S5. Cell morphology and proliferation analysis of MCF7 VEC and MCF7 MAGI1 KO cells exposed to different drugs. (**A**) Cell morphology of MCF7 VEC and MCF7 MAGI1 KO cells after the exposure to different drugs (cisplatin, olaparib, cisplatin with olaparib, alpelisib, and alpelisib with fulvestrant). (**B**) Real-time growth curve showing MCF7 VEC and MCF7 MAGI1 KO cells treated with cisplatin for 120 h (top) and graph with the analysis of the end timepoint confluency (bottom) (ns indicates not significant $p > 0.05$). (**C**) Efficacy values of cisplatin, olaparib and cisplatin + olaparib treatments on MCF7 VEC and MCF7 MAGI1 KO cells at end timepoint (120 h) relative to no treatment controls.

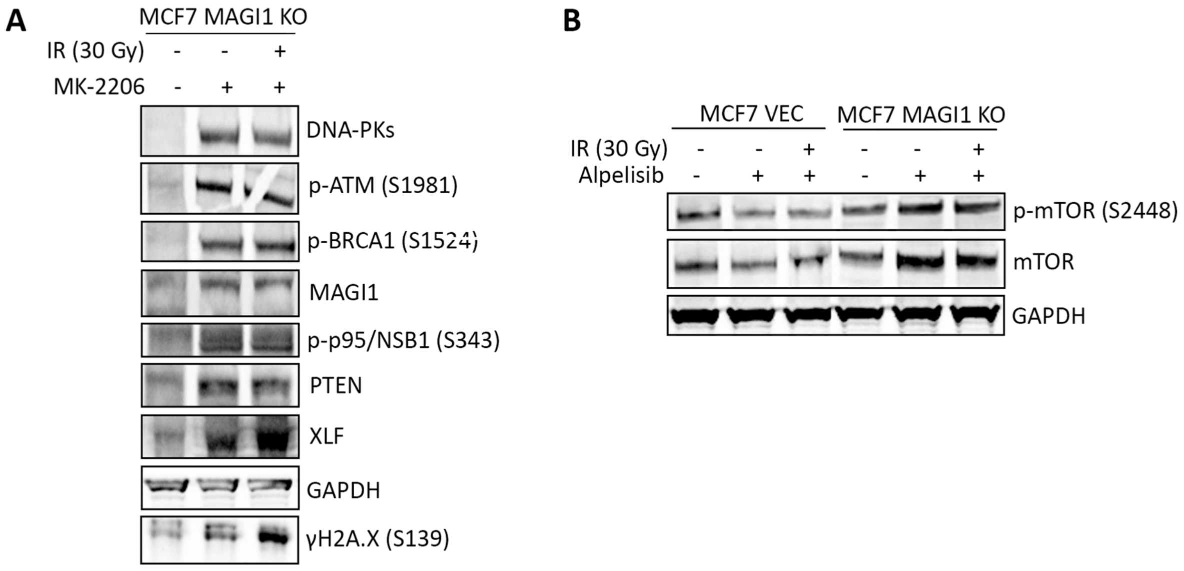

Figure S6. Western blot analysis of multiple proteins after exposure to different drugs and/or IR. (**A**) Western blot analysis of main proteins implicated in DNA repair after treatment with the AKT inhibitor MK-2206 in MCF7 MAGI1 KO cells. (**B**) Western blot analysis of total and phosphorylated levels of mTOR after exposure to IR and alpelisib in MCF7 VEC and MCF7 MAGI1 KO cells.

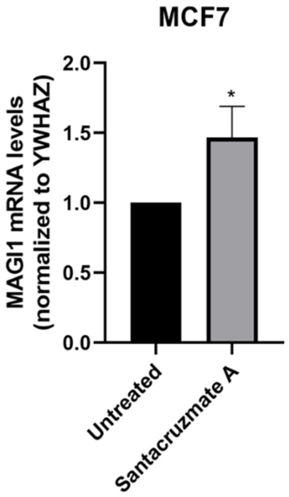

Figure S7. HDAC2 inhibition in MCF7 cells increase *MAGI1* mRNA expression levels (n = 2 independent experiments; * $p \leq 0.05$).
